# Multi-Class Classification and Multi-Output Regression of Three-Dimensional Objects Using Artificial Intelligence Applied to Digital Holographic Information

**DOI:** 10.3390/s23031095

**Published:** 2023-01-17

**Authors:** Uma Mahesh R N, Anith Nelleri

**Affiliations:** School of Electronics Engineering, Vellore Institute of Technology (VIT), Chennai 600127, Tamilnadu, India

**Keywords:** 3D information sensing, 3D vision, convolutional neural network (CNN), digital holography, machine learning, artificial intelligence (AI), supervised learning, 3D object classification, 3D object regression

## Abstract

Digital holographically sensed 3D data processing, which is useful for AI-based vision, is demonstrated. Three prominent methods of learning from datasets such as sensed holograms, computationally retrieved intensity and phase from holograms forming concatenated intensity–phase (whole information) images, and phase-only images (depth information) were utilized for the proposed multi-class classification and multi-output regression tasks of the chosen 3D objects in supervised learning. Each dataset comprised 2268 images obtained from the chosen eighteen 3D objects. The efficacy of our approaches was validated on experimentally generated digital holographic data then further quantified and compared using specific evaluation matrices. The machine learning classifiers had better AUC values for different classes on the holograms and whole information datasets compared to the CNN, whereas the CNN had a better performance on the phase-only image dataset compared to these classifiers. The MLP regressor was found to have a stable prediction in the test and validation sets with a fixed EV regression score of 0.00 compared to the CNN, the other regressors for holograms, and the phase-only image datasets, whereas the RF regressor showed a better performance in the validation set for the whole information dataset with a fixed EV regression score of 0.01 compared to the CNN and other regressors.

## 1. Introduction

Multi-class classification and multi-output regression tasks [1] are the deep learning applications that produce a single output for multiple inputs. These supervised learning techniques [2] play a vital role in the development of artificial intelligence systems in which decision making is done through discrete and continuous labels by considering multiple inputs based on the criteria of the problem at hand. Studies have emerged in the areas of learning and decision making that used multi-class classification and multi-output regression tasks such as Alzheimer’s disease classification [3,4,5,6], food ingredient classification [7], river quality prediction [8], natural gas demand forecasting [9], drug efficacy prediction [10], prediction of the audio spectrum of wind noise (represented by several sound pressure variables) of a given vehicle component [11], real-time prediction of multiple gas tank levels of a Linz–Donawitz converter gas system [12], simultaneous estimation of different biophysical parameters from remote sensing images [13], and channel estimation via the prediction of several received signals [14]. However, these real-world problems [11,12,13,14] still face major challenges such as the absence of feature/target values and the presence of noise due to the complexity of real domains. Despite dealing with these challenges, it has been proven that multi-output regression methods have a better predictive performance and computational efficiency [15]. Therefore, in the present work, we studied the implications of multi-class classification and multi-output regression tasks on 3D objects by using deep neural networks to develop intelligent and pragmatic three-dimensional (3D) vision systems. The design and development of such robust systems involve the need for efficient 3D object data-acquisition systems, 3D object reconstruction techniques from the sensed data, and the development of prudent algorithms to process the 3D information on a real-time basis. In this context, digital holography [16,17,18,19] is a potential technique for the digital sensing and computational retrieval of 3D information in a real-time process. Several applications have been demonstrated by researchers in information processing [20,21,22]. Digital holography is a coherent imaging technique in which optically generated holograms are digitally detected by a CCD/CMOS sensor and thereafter numerically reconstructed to retrieve the whole information from the optical wavefront. Thus, the stored information in the complete 3D volume of the object in the digital hologram can be numerically processed to obtain a digital complex-valued image that is a quantitative measure of the intensity and phase of the sensed complex object’s wavefront [23]. The unwrapped phase images [24] carry the quantitative depth features and are significantly important in gathering the 3D information and in processing [20,21]. In our approach, we thus demonstrated five-class classification and regression tasks of the 3D objects addressed using holograms, concatenated intensity–phase information, and phase-only holographic information via deep convolutional neural network (CNN) learning. The CNN is one of the major deep neural networks and a basic building block of deep learning. The ability of a CNN to handle greater numbers of convolutional layers and pooling layers in the feature-extraction stage and process *n* number of neurons in the classification-layer stage has made its use feasible for different kinds of tasks such as classification, autofocusing, fringe pattern denoising, image segmentation, image super-resolution, and hologram reconstruction in digital holography [25,26,27,28,29,30,31,32,33,34,35,36,37,38,39,40,41,42,43]. A CNN [44] consists of feature-extraction and classification layers. The feature-extraction layer has *n* number of convolutional and pooling layers. The classification layer is composed of a dense layer and an output layer. The convolutional layer incorporates convolutional kernels to perform convolution operations; the output of a convolution is passed through a non-linear activation function. The number of kernels and the size of the kernel in the convolutional layer are specified by the user according to the corresponding task at hand. The dense layer consists of multiple neurons, while the output layer can contain multiple neurons or a single neuron depending on the application at hand.

Kim et al. proposed a VGG 19-layer neural network to perform the hologram classification of unlabeled living cells [45]. Lam et al. performed invariant hologram classification of deformable objects using a deep CNN [46]. Zhu et al. proposed a deep learning method for the classification of microplastics using digital holograms [47]. Reddy et al. proposed deep-learning-based binary classification of 3D objects for a holographic phase-only image dataset [48]. Shimobaba et al. performed deep-CNN-based multi-class classification of data pages for holographic memory [29]. Jo et al. proposed a deep CNN to perform the holographic image classification of unlabeled living cells for anthrax and non-anthrax spores [37]. Ren et al. demonstrated the autofocusing problem as a regression task in digital holography using a deep CNN for an amplitude and phase-only hologram dataset at different recording distances [28]. In the present work, five-class classification and regression tasks using a deep CNN applied to holograms, reconstructed intensity and phase information combined in a single image, and phase (depth)-only image datasets of eighteen 3D objects are proposed. The advantage of this work compared to the previous works [28,29,37,45,46,47,48,49] was that the five-class classification and regression tasks were performed on datasets of holograms, concatenated intensity–phase images, and phase-only images of eighteen 3D objects. These three datasets of eighteen 3D objects were constructed by using recorded digital holograms and a complex-wave retrieval method [50]. Off-axis digital Fresnel holograms of 3-D objects are formed by recording the off-axis geometry at different distances; by post-processing the holograms, the datasets of the concatenated intensity–phase images and phase-only images were obtained. These three datasets were further passed through the deep CNN to perform five-class classification and regression tasks. The five-class classification and regression tasks in supervised learning applied to the digital holographic information of the eighteen 3D objects using a deep CNN were equivalent to 3D object allocation and prediction performed on the digital holographic datasets that produced discrete and continuous labels as output, which justified the rationale behind the present work. The CNN was trained on all three datasets separately to generate the results. For the five-class classification, results such as loss/accuracy graphs of the training/validation sets, a confusion matrix, performance metrics, receiver operating characteristics (ROC), and precision-recall characteristics are shown for the validation of the present work. Similarly for the five-class regression task, the results such as loss, mean square error (MSE), and mean absolute error (MAE) curves were plotted for both the training/validation sets; and performance metrics such as the MAE, R^2^ score (coefficient of determination), and explained-variance (EV) regression score for the test/validation sets are shown for the confirmation of the work. Further, the CNN was compared with machine learning classifiers and regressors such as KNN, MLP, DT, RF, and ET separately using all three datasets for both of the tasks. The proof of the proposed concept was demonstrated using a real-time off-axis digital holographic experiment for sensing and retrieving the 3D object information, which was further processed using AI/ML techniques.

## 2. Theory

In this section, we describe the digital hologram sensing, retrieval, and processing of the 3D information using the deep CNN and machine learning algorithms.

### 2.1. Sensing and Retrieval of 3D Information of the Objects Using Off-Axis Digital Fresnel Holography

**Figure 1:**
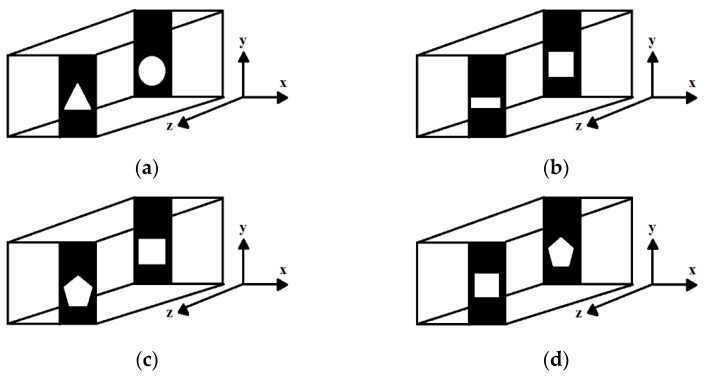
The 3D objects used in the off-axis digital holographic recording geometry: (**a**) *circle–triangle*; (**b**) *square–rectangle*; (**c**) *square–pentagon*; (**d**) *pentagon–square.* Circle: 2 mm in diameter; triangle: 2 mm in x and y directions; square: 2 mm in x and y directions; pentagon: 2 mm in x and y directions; rectangle: 2 mm in x direction and 1 mm in y direction. The distance between the first plane and second plane was 8 mm in the z direction.

The construction and modeling of the 3D objects are shown in Section S1.1 of the Supplementary Materials. Figure 1 shows the schematic diagrams of four of the eighteen 3D objects used for the recording of the off-axis digital Fresnel holograms.

The 3D objects shown in Figure 1 consisted of two different planes; namely, the first plane and the second plane, which had different features that were separated by a distance of z=8 mm. The construction of the remaining fourteen 3D objects was similar to that of the four 3D objects shown in Figure 1 but with different features on each plane [48]. In total, eighteen 3D objects were considered for the proposed five-class classification and regression tasks [49]. The 3D objects were characterized by their intensity and phase information. In the construction shown in Figure 1, when light passed through the first plane, the amplitude and phase information of the object with the features of the first plane were obtained. Then, after propagating by a distance of z using free space propagation, the amplitude and phase information of the next object features in the second plane were also obtained.

**Figure 2:**
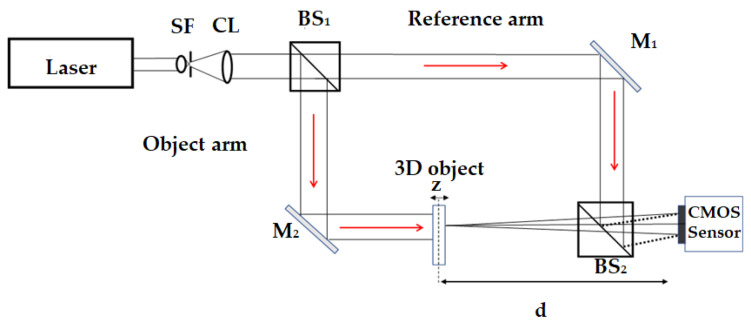
Off-axis digital holographic recording geometry used for the recording of holograms of 3D objects. SF: spatial filter assembly; CL: collimation lens; BS: beam splitter; M: mirror; CMOS: camera sensor.

Section S1.2 presents the details of the digital recording and numerical reconstruction of the holograms to obtain the complex 3D object wave information. Figure 2 describes the experimental setup (of the Mach–Zehnder digital holographic recording geometry in an off-axis scheme) used for the recording of the holograms of the 3D objects [49]. A He-Ne laser source with a wavelength λ=632.8 nm was used here. The holograms were recorded by using a CMOS sensor with a square pixel pitch of 6 μm×6 μm at an interference angle of θ=1.4°. The size of each recorded hologram was 1600×1600. Next, the complex-wave retrieval method [50] was applied to the recorded holograms of the 3D objects to obtain the complex-wave fields of the objects at the recording plane. Further, an inverse Fresnel transform was applied on the retrieved complex-wave field to obtain a 2D digital complex-valued image at the object plane. The 2D digital complex-valued images contained 3D information in the form of the intensity and phase.

The intensity and phase information present in the 2D digital complex-valued images were extracted and further united via the method of concatenation to form concatenated intensity–phase (whole information) images, and phase (depth)-only information was also extracted from the 2D digital complex-valued images to form phase images. The off-axis Mach–Zehnder holographic geometry was suitable for both transmitting and reflecting the types of objects. The object beam arm could be modified appropriately for the reflective objects or specimens. In the present paper, we modeled the 3D objects to use them in transmission mode to demonstrate the proof of concept of the proposed application.

### 2.2. Multi-Class Classification and Multi-Output Regression of 3D Objects Using Holographic Information

Section S1.3 shows the equations that governed the 3D object set formation of the datasets of the sensed holograms, concatenated intensity–phase images, and phase-only images. The holographic information of 3D objects can be processed using an AI-based approach in several ways. One method is to apply direct learning from the sensed hologram data. Another method is to learn the retrieved 3D object information from digital holograms; i.e., by forming a dataset of the retrieved intensities and phases combined to form concatenated intensity–phase images. Since the phase information contains the depth features, a phase-only information or phase-only image dataset can be learned to accomplish the tasks. In the present paper, we addressed the above approaches for the multi-class classification and multi-output regression tasks of the 3D objects in supervised learning by using a deep CNN and comparing the results with those of standard machine learning algorithms. The five-class classification and regression tasks in supervised learning applied to the digital holographic information of eighteen 3D objects using a deep CNN was equivalent to 3D object allocation and prediction performed on the digital holographic datasets that produced discrete and continuous labels as output, which justified the rationale behind the present work. The eighteen 3D objects considered for the above problem were classified into five different sub-classes (Class-a, Class-b, Class-c, Class-d, and Class-e) to perform the five-class classification and regression tasks using the following equations:

(1) $$\mathrm{Class-a}:\;\left\{T_{1}\right\}\;\in \left\{a_{d}{}_{i},\;b_{d}{}_{i},c_{d}{}_{i},e_{d}{}_{i}\right\}$$

(2) $$\mathrm{Class-b}:\;\left\{T_{2}\right\}\in \left\{\;f_{d}{}_{i},g_{d}{}_{i},h_{d}{}_{i},k_{d}{}_{i}\right\}$$

(3) $$\mathrm{Class-c}:\;\left\{T_{3}\right\}\in \left\{l_{d}{}_{i},m_{d}{}_{i},n_{d}{}_{i},\;o_{d}{}_{i}\right\}$$

(4) $$\mathrm{Class-d}:\;\left\{T_{4}\right\}\in \left\{\;\;p_{d}{}_{i},q_{d}{}_{i},r_{d}{}_{i}\right\}$$

(5) $$\mathrm{Class-e}:\;\left\{T_{5}\right\}\in \left\{s_{d}{}_{i},\;t_{d}{}_{i},u_{d}{}_{i}\right\}$$

where ‘$d_{i}$’ represents the distance between the recording plane and object plane, and i denotes the indices of the individual objects. The combined objects *circle–pentagon*$\;\left(a_{d}{}_{i}\right)$, *circle–triangle*$\left(b_{d}{}_{i}\right)$, *circle–square* ($c_{d}{}_{i}$), and *circle–rectangle* ($e_{d}{}_{i}$) were considered for Class-a. The combined objects *square–circle* ($f_{d}{}_{i}$), *square–triangle* ($g_{d}{}_{i}$), *square–rectangle* ($h_{d}{}_{i}$), and *square–pentagon* ($k_{d}{}_{i}$) were considered for Class-b. The combined objects *triangle–circle* ($l_{d}{}_{i}$), *triangle–square* ($m_{d}{}_{i}$), *triangle–rectangle* ($n_{d}{}_{i}$), and *triangle–pentagon* ($o_{d}{}_{i}$) were considered for Class-c. The combined objects *pentagon–circle* ($p_{d}{}_{i}$), *pentagon–square* ($q_{d}{}_{i}$), and *pentagon–triangle* ($r_{d}{}_{i}$) were considered for Class-d. Finally, the combined objects *rectangle–circle* ($s_{d}{}_{i}$), *rectangle–square* ($t_{d}{}_{i}$), and *rectangle–triangle* ($u_{d}{}_{i}$) were considered for Class-e. The five-class classification and regression tasks of the 3D objects using the hologram dataset are shown in Equations (1)–(5). The five-class classification and regression tasks for the concatenated intensity–phase (whole information) image dataset were performed by using the following Equations (6)–(10), respectively.

(6) $$\mathrm{Class-a}:\;\left\{RT_{INPH1}\right\}\;\in \left\{Ra_{d}{}_{i,INPH},\;Rb_{d}{}_{i,INPH},Rc_{d}{}_{i,INPH},Re_{d}{}_{i,INPH}\right\}$$

(7) $$\mathrm{Class-b}:\;\left\{RT_{INPH2}\right\}\in \left\{Rf_{d}{}_{i,INPH},Rg_{d}{}_{i,INPH},Rh_{d}{}_{i,INPH},Rk_{d}{}_{i,INPH}\right\}$$

(8) $$\mathrm{Class-c}:\;\left\{RT_{INPH3}\right\}\in \left\{Rl_{d}{}_{i,INPH},Rm_{d}{}_{i,INPH},Rn_{d}{}_{i,INPH},\;Ro_{d}{}_{i,INPH}\right\}$$

(9) $$\mathrm{Class-d}:\;\left\{RT_{INPH4}\right\}\in \left\{Rp_{d}{}_{i,INPH},Rq_{d}{}_{i,INPH},Rr_{d}{}_{i,INPH}\right\}$$

(10) $$\mathrm{Class-e}:\;\left\{RT_{INPH5}\right\}\in \left\{Rs_{d}{}_{i,INPH},\;Rt_{d}{}_{i,INPH},Ru_{d}{}_{i,INPH}\right\}$$

The five-class classification and regression tasks of the phase (depth)-only image dataset were performed by using Equations (11)–(15), respectively.

(11) $$\mathrm{Class-a}:\;\left\{RT_{PH1}\right\}\;\in \left\{Ra_{d}{}_{i,PH},Rb_{d}{}_{i,PH},Rc_{d}{}_{i,PH},Re_{d}{}_{i,PH}\right\}$$

(12) $$\mathrm{Class-b}:\;\left\{RT_{PH2}\right\}\in \left\{Rf_{d}{}_{i,PH},Rg_{d}{}_{i,PH},Rh_{d}{}_{i,PH},Rk_{d}{}_{i,PH}\right\}$$

(13) $$\mathrm{Class-c}:\;\left\{RT_{PH3}\right\}\in \left\{Rl_{d}{}_{i,PH},Rm_{d}{}_{i,PH},Rn_{d}{}_{i,PH},\;Ro_{d}{}_{i,PH}\right\}$$

(14) $$\mathrm{Class-d}:\;\left\{RT_{PH4}\right\}\in \left\{Rp_{d}{}_{i,PH},Rq_{d}{}_{i,PH},Rr_{d}{}_{i,PH}\right\}$$

(15) $$\mathrm{Class-e}:\;\left\{RT_{PH5}\right\}\in \left\{Rs_{d}{}_{i,PH},\;Rt_{d}{}_{i,PH},Ru_{d}{}_{i,PH}\right\}$$

The deep CNN was used to perform the five-class classification and regression tasks by employing datasets of holograms, concatenated intensity–phase images, and phase-only images. Further, the five-class classification and regression tasks for different digital holographic datasets performed using the deep CNN were compared via machine learning algorithms such as the K-nearest neighbor (KNN), multi-layer perceptron (MLP), decision tree (DT), random forest (RF), and extra trees (ET). In this way, the five-class classification and regression tasks were performed for the different digital holographic datasets using deep learning and machine learning frameworks.

## 3. Architecture of CNN for Multi-Class Classification and Multi-Output Regression

**Figure 3:**
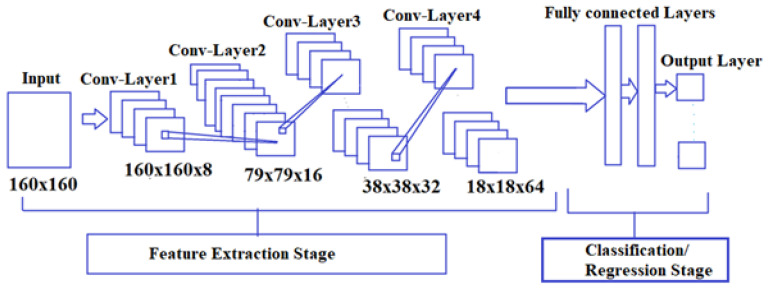
Block diagram of CNN for multi-class classification and multi-output regression.

Figure 3 shows a block diagram of the CNN that was used to perform the five-class classification and regression tasks for the different digital holographic information, which consisted of datasets of holograms, concatenated intensity–phase (whole information) images, and phase (depth)-only images; i.e., the CNN took the inputs from all three datasets independently. Section S2.1 provides the details of the mathematical model of the CNN used for the multi-class classification and multi-output regression.

Figure 3 describes the architecture of the CNN, which contained four convolutional and four pooling layers, fully connected layers, and an output layer. The classification stage was used for the five-class classification and regression purposes. In the five-class regression, the classification stage was modified into the regression stage to implement the task. The convolutional layer operated on the raw input and the kernel to generate the output, which was then further processed by the pooling layer. Here, each convolutional layer consisted of the rectified linear unit (ReLU) activation function. The number of kernels was equal to 8 in the first convolutional layer, 16 in the second, 32 in the third, and finally 64 in the fourth. The kernel size was equal to three in all convolutional layers. The pooling layer accepted the input from the convolutional layer to minimize the dimensionality of the feature map. The pooling technique used here was MaxPooling2D. The pooling layer did not affect the number of parameters because it only reduced the dimensionality of the feature map. After four successive stages of convolution and the pooling operations in the feature-extraction stage, the final pooling-stage output was given to the classification stage to perform the five-class classification and regression tasks. The fully connected layer took the input from the fourth pooling stage; i.e., the 2D data, and converted it into 1D data through the flattened layer before processing it through the fully connected layer. In the fully connected layer, the number of neurons considered was 16. The output layer received the input from the fully connected layer to perform the five-class classification and regression tasks. For the five-class classification, the softmax function was used; for regression, the linear function was used in the output layer. The number of neurons in the output layer for the five-class classification and regression tasks considered was five. The summary of the proposed deep CNN model used for five-class classification and regression tasks is shown in Table 1.

Section S2.2 provides the details on the performance metrics used for the multi-class classification and multi-output regression tasks.

## 4. Dataset Preparation

**Figure 4:**
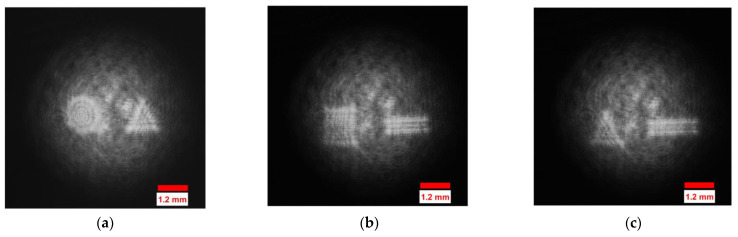

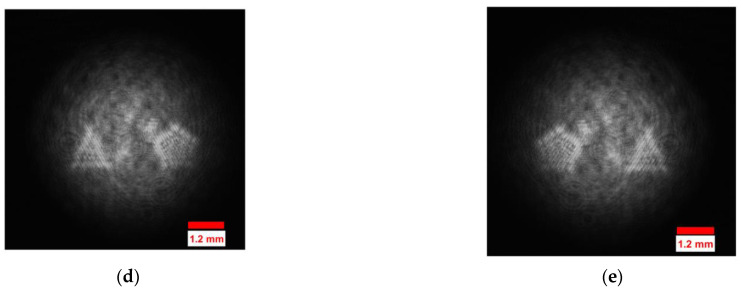
Representative images of the digital holograms of 3D objects recorded at distance of $d_{3}=200$ mm: (**a**) *circle–triangle* ($b_{d_{3}}$); (**b**) *square–rectangle* ($h_{d_{3}}$); (**c**) *triangle–rectangle* ($n_{d_{3}}$); (**d**) *triangle–pentagon* ($o_{d_{3}}$); (**e**) *pentagon–triangle* ($r_{d_{3}}$).

The datasets of the holograms, concatenated intensity–phase images, and phase (depth)-only images were created using the eighteen 3D objects considered in this study. Digital holograms of the eighteen 3D objects were created using Mach–Zehnder off-axis digital holographic geometry as shown in Figure 2. The 18 three-dimensional objects were used in the off-axis geometry to form 63 holograms with a size of 1600×1600 using a CMOS sensor at 15 different distances of $d_{1}=180$ mm, $d_{2}=185$ mm, $d_{3}=200$ mm, $d_{4}=201$ mm, $d_{5}=205$ mm, $d_{6}=210$ mm, $d_{7}=220$ mm, $d_{8}=250$ mm, $d_{9}=251$ mm, $d_{10}=255$ mm, $d_{11}=300$ mm, $d_{12}=305$ mm, $d_{13}=310$ mm, $d_{14}=311$ mm, and $d_{15}=315$ mm. Figure 4 shows the digital holograms of five of the 3-D objects recorded at a distance of $d_{3}=200$ mm.

**Figure 5:**
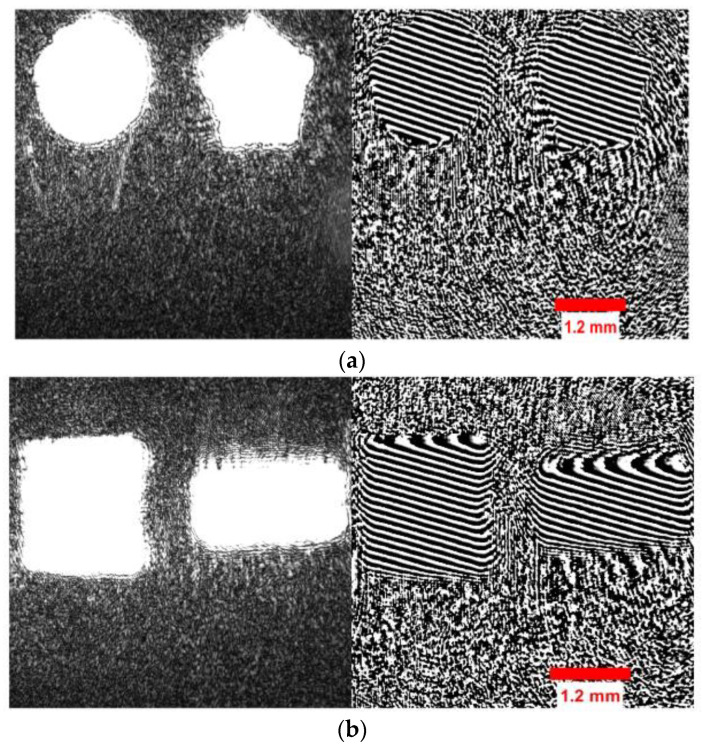
(**a**) Concatenated intensity–phase image of the circle–pentagon ($Ra_{d}{}_{1,INPH})$ object that belonged to Class-a; (**b**) concatenated intensity–phase image of the square–rectangle ($Rh_{d}{}_{1,INPH}$) object that belonged to Class-b.

**Figure 6:**
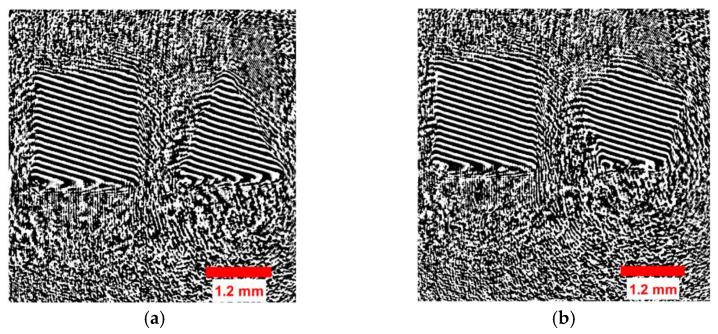
Reconstructed phase images at a distance of $d_{1}=180\;\mathrm{mm}$: (**a**) square–triangle ($Rg_{d_{1,PH}}$); (**b**) square–pentagon ($Rk_{d_{1,PH}}$).

The holograms of eighteen 3D objects were used to obtain 2D digital complex-valued images using the complex-wave retrieval method [50]. The datasets of the holograms, concatenated intensity–phase images, and phase-only images were formed by rotating each image in the respective datasets by 5°. Then, these three digital holographic datasets, which consisted of 2268 images separately, were passed through the deep CNN independently as shown in Figure 3 to perform the five-class classification and regression tasks. The hologram and phase-image size considered for the input of the CNN was 160×160 from 1600×1600. The concatenated intensity–phase image size considered for the input of the CNN was 160×160 from 1600×3200. The five-class classification and regression tasks of the hologram dataset were governed by Equations (1)–(5), respectively. The five-class classification and regression tasks of the concatenated intensity–phase image dataset were governed by Equations (6)–(10). Figure 5a shows the concatenated intensity–phase image of the object ‘*circle–pentagon*’ ($Ra_{d}{}_{1,INPH}$) present in Class-a at a distance of $d_{1}=180\;\mathrm{mm}$. Similarly, Figure 5b shows the concatenated intensity–phase image of the object ‘*square–rectangle*’ ($Rh_{d}{}_{1,INPH}$) present in Class-b at a distance of $d_{1}=180\;\mathrm{mm}$. Figure 6a shows the reconstructed phase image $\left(Rg_{d_{1,PH}}\right)$ of the object ‘*square–triangle*’ present in Class-b at a distance of $d_{1}=180\;\mathrm{mm}$. Figure 6b shows the reconstructed phase image $\left(Rk_{d_{1,PH}}\right)$ of the object ‘*square–pentagon*’ present in Class-b at a distance of $d_{1}=180\;\mathrm{mm}$. The five-class classification and regression tasks of the phase-only image dataset were also governed by Equations (11)–(15), respectively. Next, after performing the five-class classification and regression for the datasets of the holograms, concatenated intensity–phase images, and phase-only images separately using the deep CNN, the results were further correlated with those of machine learning algorithms such as KNN, MLP, DT, RF, and ET. The number of nearest neighbors considered for the KNN classifier and regressor was k=5.

The MLP classifier and regressor, which consisted of a single hidden layer with ReLU as the activation function, were trained using an Adam optimizer with a learning rate and regularization rate (α) of 0.0003. The DT classifier and regressor was trained by setting max_depth=2. The RF classifier and regressor was trained by setting n_estimators=10, max_depth=none, and min_samples_split=2. The ET classifier and regressor were also trained similarly to the RF classifier and regressor using the same parameters. For the five-class classification and regression tasks, all three digital holographic datasets were separated into training, validation, and test sets with respective proportions of **75:15:10**. The training set consisted of 340 images in Class-a, Class-b, Class-c, and Class-d; and 341 images in Class-e. The validation set consisted of 68 images each in all five classes. The test set consisted of 45 images each in Class-a, Class-b, Class-c, and Class-d; and 47 images in Class-e. For the five-class classification, the deep CNN was trained for 100 epochs using an Adam optimizer whose learning rate considered was 0.0003; categorical-cross entropy was used as a loss function. Similarly to the five-class regression, the training of the deep CNN was performed like that for the five-class classification with the mean square error (MSE) as the loss function; the metrics considered were the mean square error (MSE) and the mean absolute error (MAE). The learning rate of the deep CNN model for both of the tasks was fixed throughout the training. The execution of the deep CNN was conducted using Python programming in TensorFlow, and the machine learning classifier and regressor execution was conducted using scikit learn.

## 5. Results and Discussion

### 5.1. Training of CNN for Multi-Class Classification on Holograms

**Figure 7:**
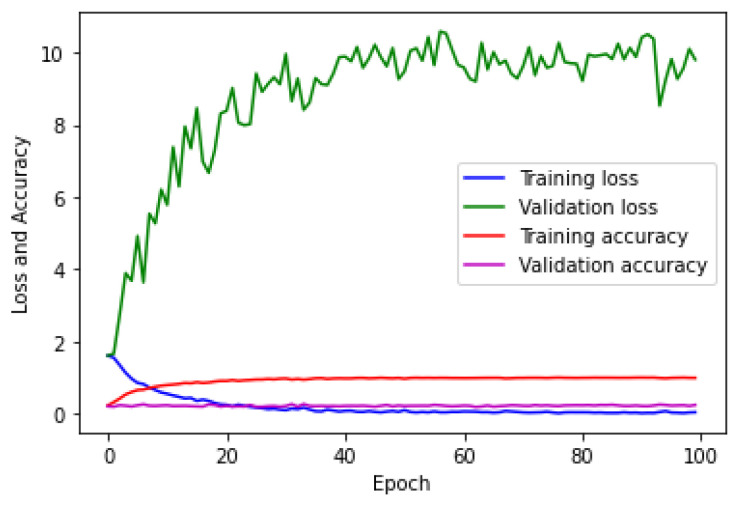
Error/accuracy plot for training/validation sets for the hologram dataset.

The CNN was trained for the hologram dataset on both the training/validation sets with a subset of 21/20 images in one epoch. The same process was repeated simultaneously with 81/17 steps for both sets in the remaining epochs. Figure 7 shows the loss/accuracy plot obtained by the CNN. Figure 7 shows that the validation error was higher than the training error and that the accuracy of the training set was greater than that of the validation set. This confirmed that the CNN did not correctly fit.

**Figure 8:**
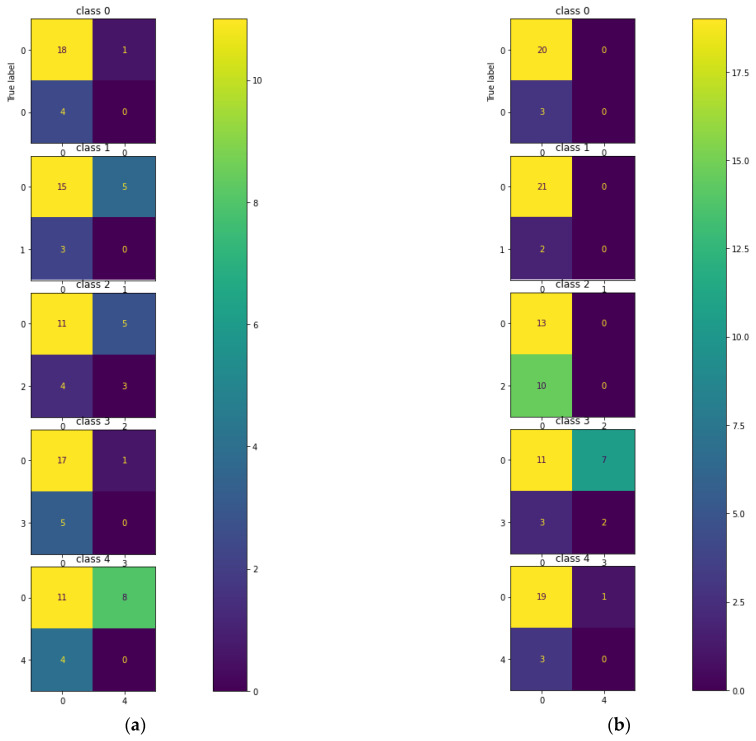

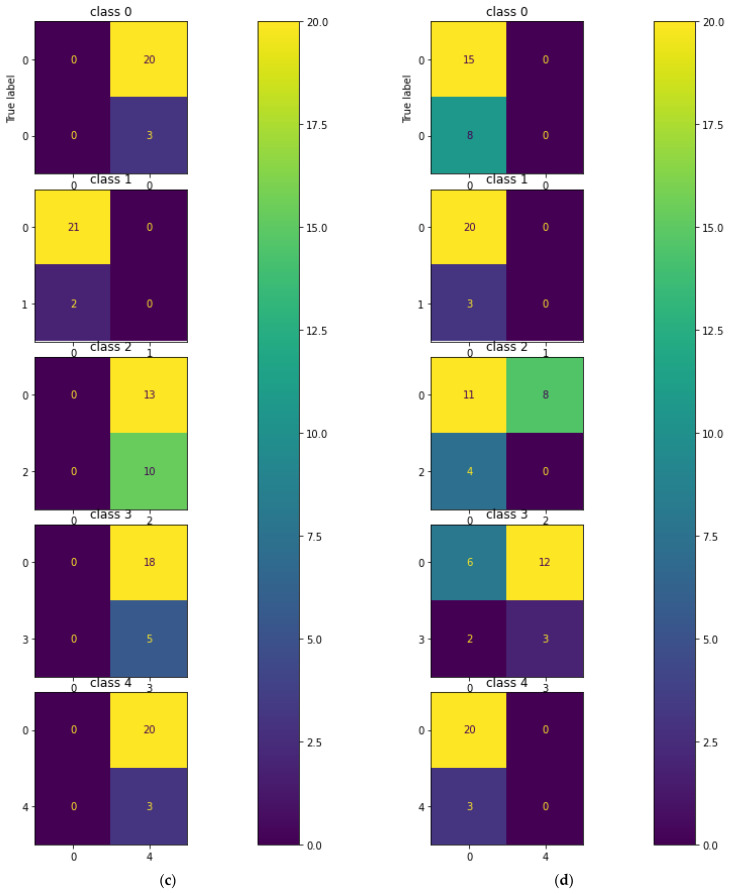

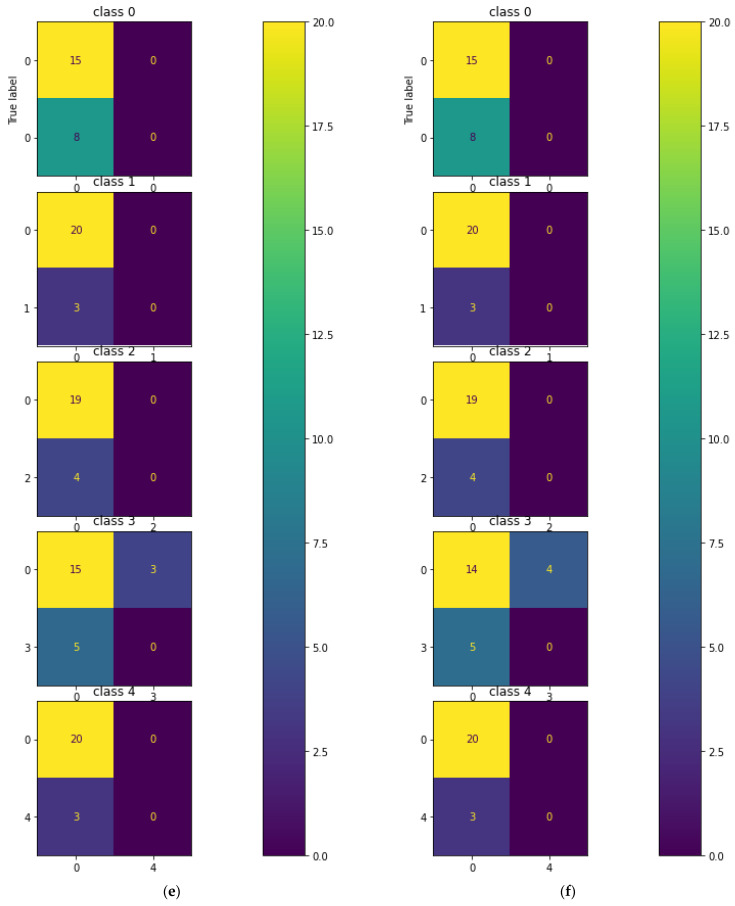
Multi-class confusion matrix for the hologram dataset: (**a**) CNN; (**b**) KNN; (**c**) MLP; (**d**) DT; (**e**) RF; (**f**) ET.

The testing of the CNN on the hologram dataset was performed separately with a batch size of 23 images. Figure 8 describes the multi-class confusion matrix obtained from the test set. Further, the performance of the multi-class classification was compared with certain machine learning classifiers such as KNN, MLP, DT, RF, and ET. The multi-class confusion matrix obtained by the KNN, MLP, DT, RF, and ET classifiers with a batch size of 23 images for the hologram dataset on the test set are also shown in Figure 8. In the figure, it can be observed that the confusion matrix was represented for multiple classes; i.e., for five classes. Section S2.3 provides the details on the general confusion matrix for the five classes.

The performance metrics obtained from the CNN, KNN, MLP, DT, RF, and ET classifiers for the hologram dataset are shown in Table 2. The metric macro average was obtained by averaging over all five classes for the respective labels. The metrics of the micro average, weighted average, and samples average were calculated using the confusion matrix. Table 2 shows that the CNN had a greater accuracy for Class-a compared to the other classes. The KNN and MLP classifiers had a greater accuracy for Class-b compared to the other classes. The DT, RF, and ET classifiers had a higher accuracy for Class-b and Class-e compared to the other classes. Table 3 shows the computational costs and complexity parameters such as the floating-point operations (FLOPs), training time, and test time for the CNN, as well as for the other machine learning classifiers (KNN, MLP, DT, RF, and ET) for the hologram dataset.

**Figure 9:**
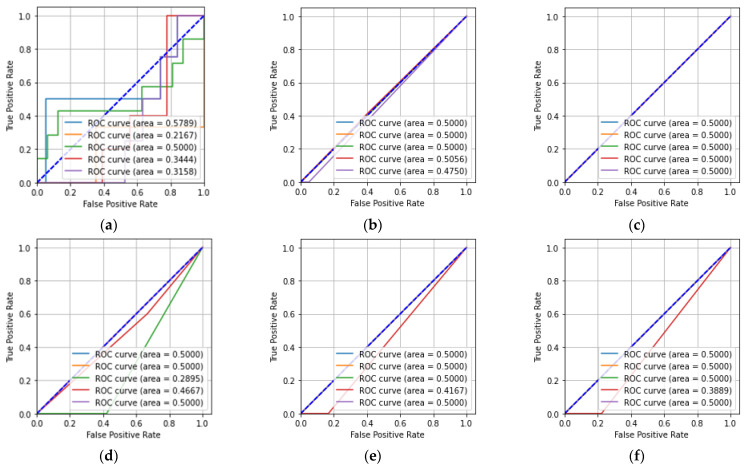
ROCs for the hologram dataset: (**a**) CNN; (**b**) KNN; (**c**) MLP; (**d**) DT; (**e**) RF; (**f**) ET.

Table 3 shows that the number of floating-point operations (FLOPs) for the CNN was greater compared to that of the other machine learning classifiers. In Table 3, it can also be seen that the training time and test time for the CNN were greater compared to the other machine learning classifiers. The receiver operating characteristic (ROC) and precision-recall characteristic were also used to describe the performance of the five-class classification task. Figure 9 shows the ROCs obtained from the CNN, KNN, MLP, DT, RF, and ET classifiers for the hologram dataset.

**Figure 10:**
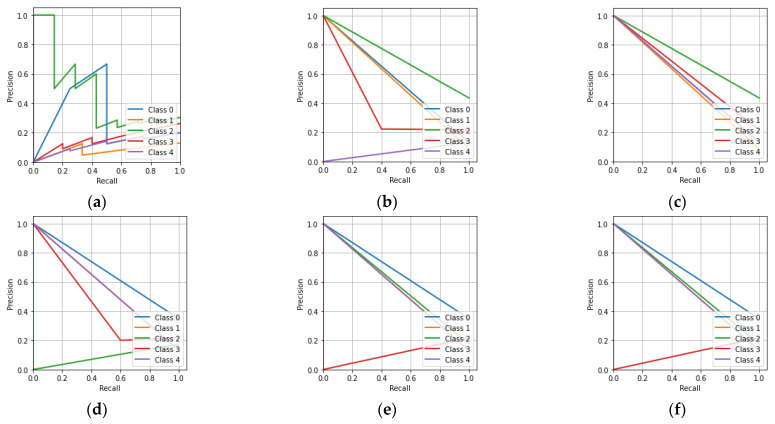
Precision-recall characteristics for the hologram dataset: (**a**) CNN; (**b**) KNN; (**c**) MLP; (**d**) DT; (**e**) RF; (**f**) ET.

In Figure 9a, it can be seen that the CNN has a better area under the curve (AUC) value of 0.57 for Class-a compared to the other classes. Similarly, it can be seen that the KNN classifier had a better AUC value for Class-d compared to the other classes. The MLP classifier had equal AUC values for all five of the classes. The DT classifier had better AUC values for Class-a, -b, and -e compared to the other classes. The RF and ET classifiers have better AUC values for Class-a, -b, -c, and -e compared to Class-d. Figure 10 describes the precision-recall characteristics obtained by the CNN, KNN, MLP, DT, RF, and ET classifiers for the hologram dataset.

Figure 10a shows that the CNN had a lower precision as the recall approached unity for all five of the classes. Similarly to the remaining precision-recall characteristics, the machine learning classifiers also had a lower precision when the recall approached unity for all five of the classes.

### 5.2. Training of CNN for Multi-Class Classification on Concatenated Intensity–Phase Image Dataset

**Figure 11:**
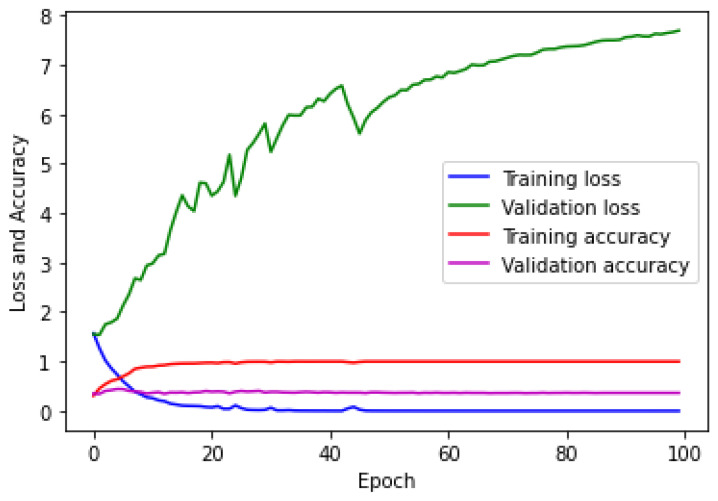
Loss and accuracy plot for the concatenated intensity–phase (IP) image dataset.

The CNN was trained on the concatenated intensity–phase image dataset in the same manner as that of the hologram dataset. The loss/accuracy plot obtained by the CNN is shown in Figure 11, which depicts that the validation error was higher than the training error and the accuracy of the training set was greater than that of the validation set. Therefore, it can be said that the CNN model was overfitting.

**Figure 12:**
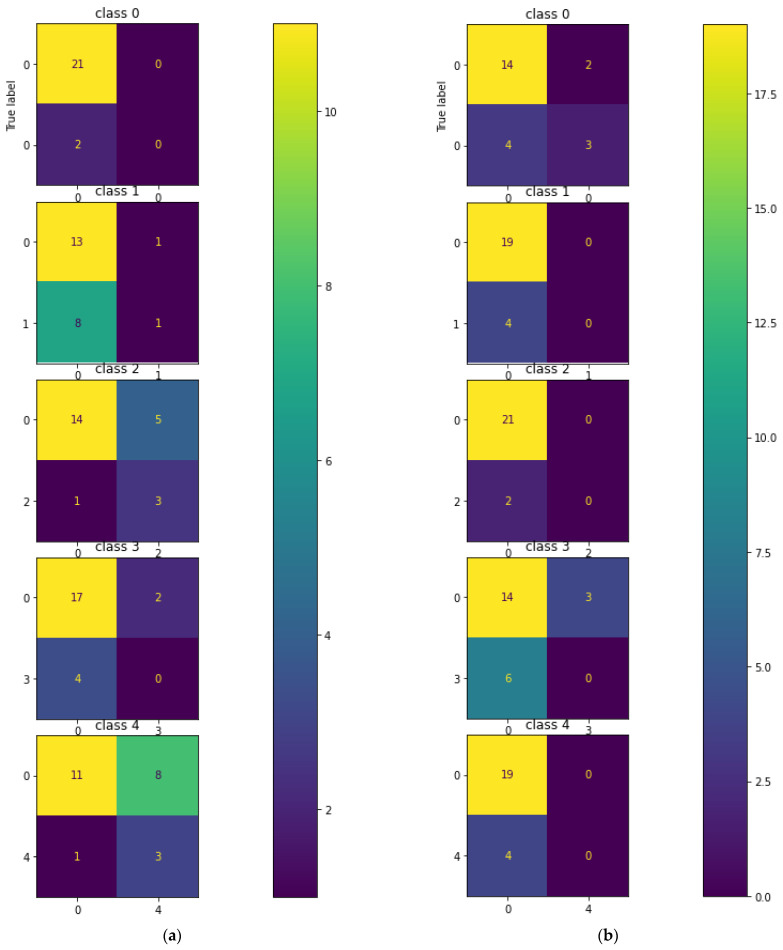

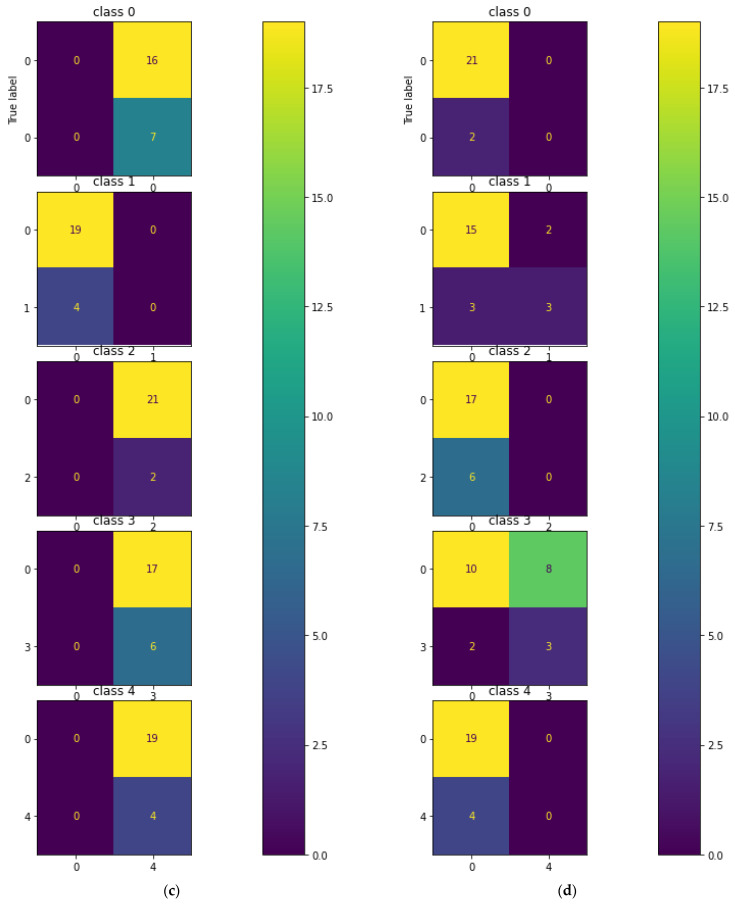

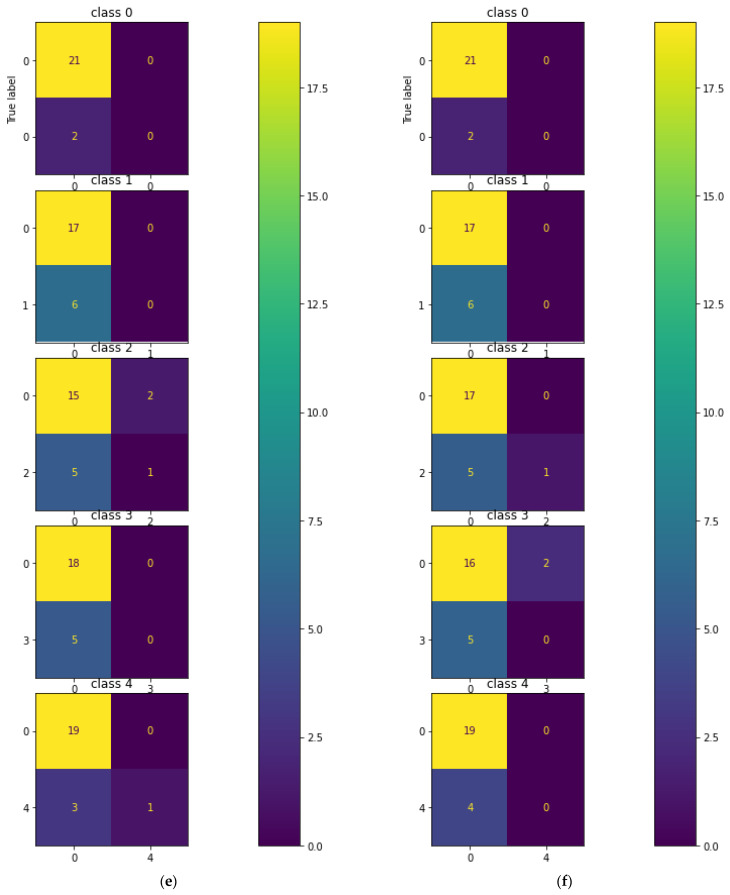
Multi-class confusion matrix for the whole information dataset: (**a**) CNN; (**b**) KNN; (**c**) MLP; (**d**) DT; (**e**) RF; (**f**) ET.

The testing of the CNN on the concatenated intensity–phase image dataset was performed in the same manner as that of the hologram dataset. The multi-class confusion matrix obtained by the CNN is shown in Figure 12. Further, the CNN was compared with the machine learning classifiers. The testing of the machine learning classifiers on the concatenated intensity–phase image dataset was performed in the same manner as that of the hologram dataset. Figure 12 shows the confusion matrix obtained by the KNN, MLP, DT, RF, and ET classifiers for all five classes for the concatenated intensity–phase image dataset.

Table 4 shows that the CNN, DT, RF, and ET classifiers had a higher accuracy for Class-a compared to that for other classes. The KNN and MLP classifiers had a greater accuracy for Class-b and -c compared to the other classes. Table 5 shows the computational costs and complexity parameters such as the floating-point operations (FLOPs), training time, and test time for the CNN and the machine learning classifiers (KNN, MLP, DT, RF, and ET) for the concatenated intensity–phase image dataset.

**Figure 13:**
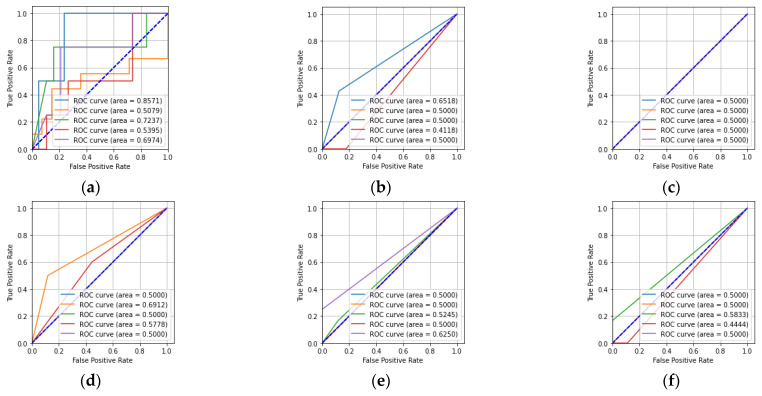
ROCs for whole information dataset: (**a**) CNN; (**b**) KNN; (**c**) MLP; (**d**) DT; (**e**) RF; (**f**) ET.

In Table 5, it can be seen that the number of floating-point operations (FLOPs) for the CNN was high compared to that of the other machine learning classifiers. Based on Table 5, it also can be said that the training time and the test time for the CNN were higher compared to those of the machine learning classifiers. Figure 13 shows the ROCs obtained from the CNN and the machine learning classifiers for all five classes for the concatenated intensity–phase image dataset.

**Figure 14:**
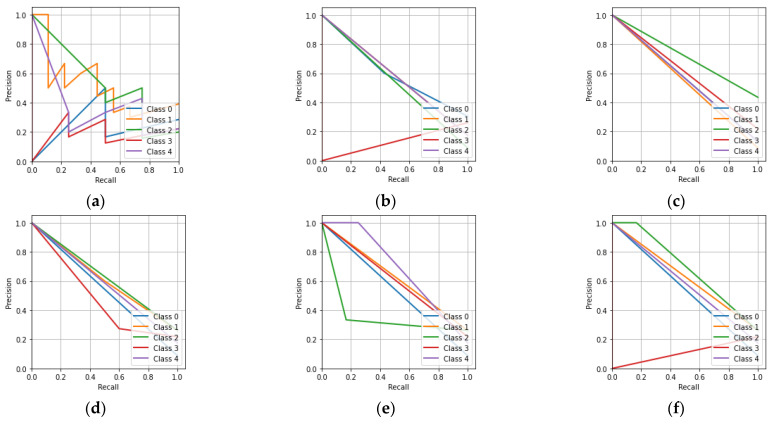
Precision-recall characteristic for the whole information dataset: (**a**) CNN; (**b**) KNN; (**c**) MLP; (**d**) DT; (**e**) RF; (**f**) ET.

Figure 13a,b shows that the CNN and KNN classifiers had higher AUC values for Class-a compared to the other classes. Similarly, it can be said that the MLP classifier had equal AUC values for all five of the classes. The DT, RF, and ET classifiers had higher AUC values for Class-b, -e, and -c compared to the other classes. Figure 14 shows the precision-recall characteristics obtained by the CNN and the machine learning classifiers for the concatenated intensity–phase image dataset.

Figure 14a shows that the CNN had a lower precision as the recall approached unity. Similarly, it can be said that the KNN, MLP, DT, RF, and ET classifiers had a lower precision as the recall approached unity.

### 5.3. Training of CNN for Multi-Class Classification on Phase-Only Information

**Figure 15:**
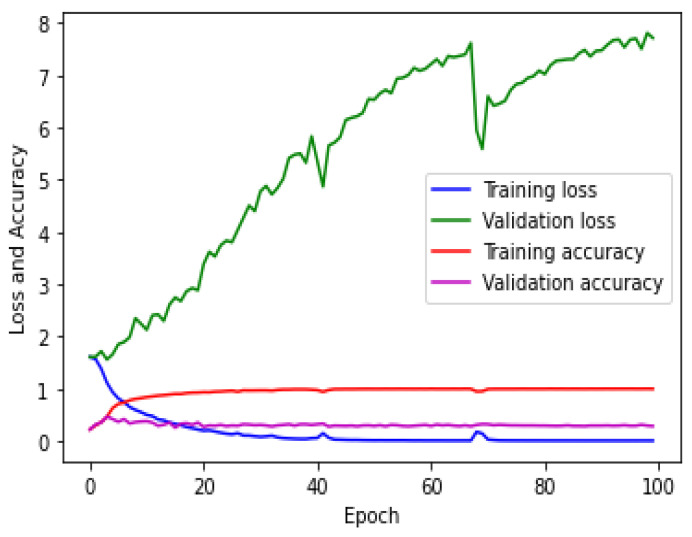
Error and accuracy measurements on training/validation sets for phase-only image dataset.

The CNN was trained on the phase-only image dataset in the same manner as that of the hologram dataset. The loss/accuracy plot for both the training/validation sets obtained by the CNN for the phase-only image dataset is shown in Figure 15.

**Figure 16:**
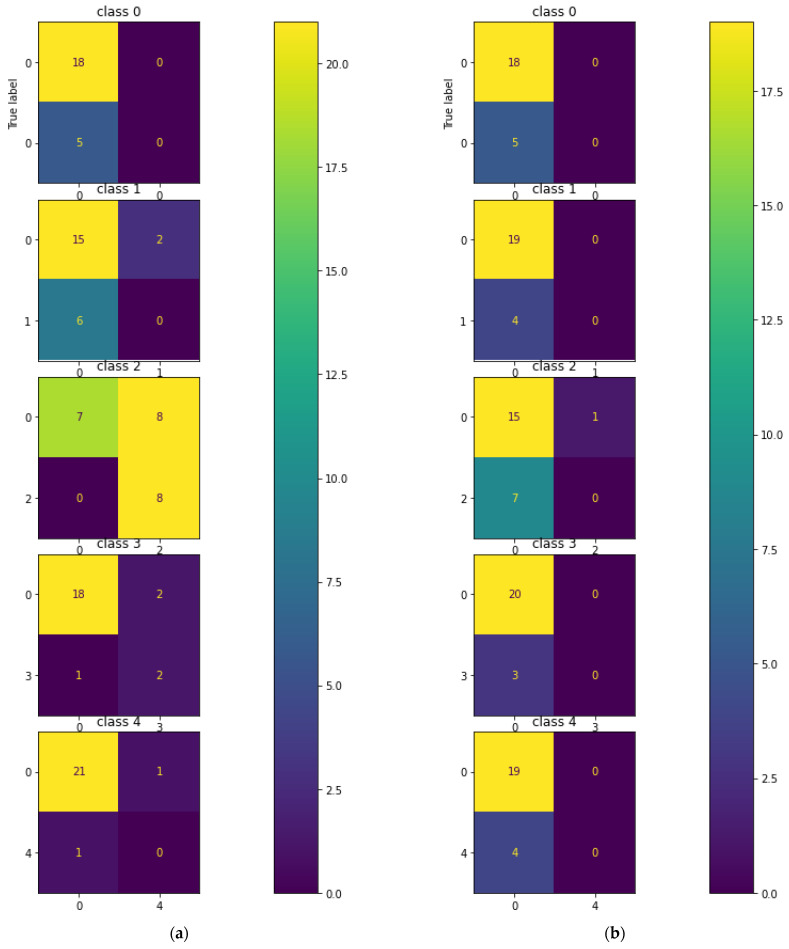

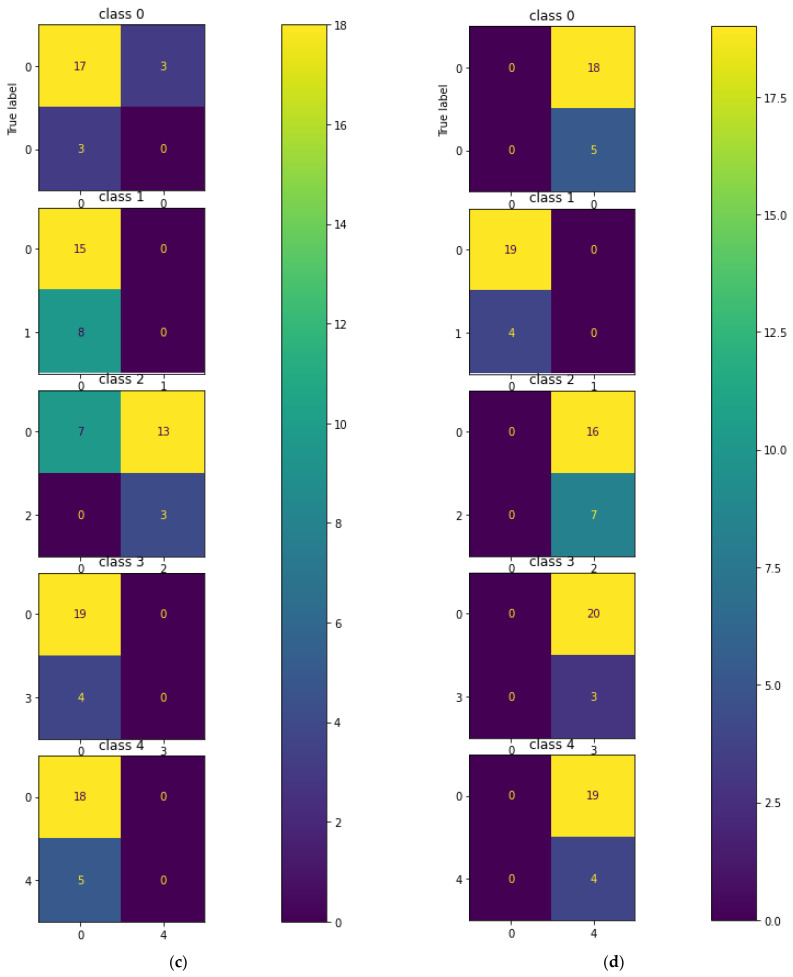

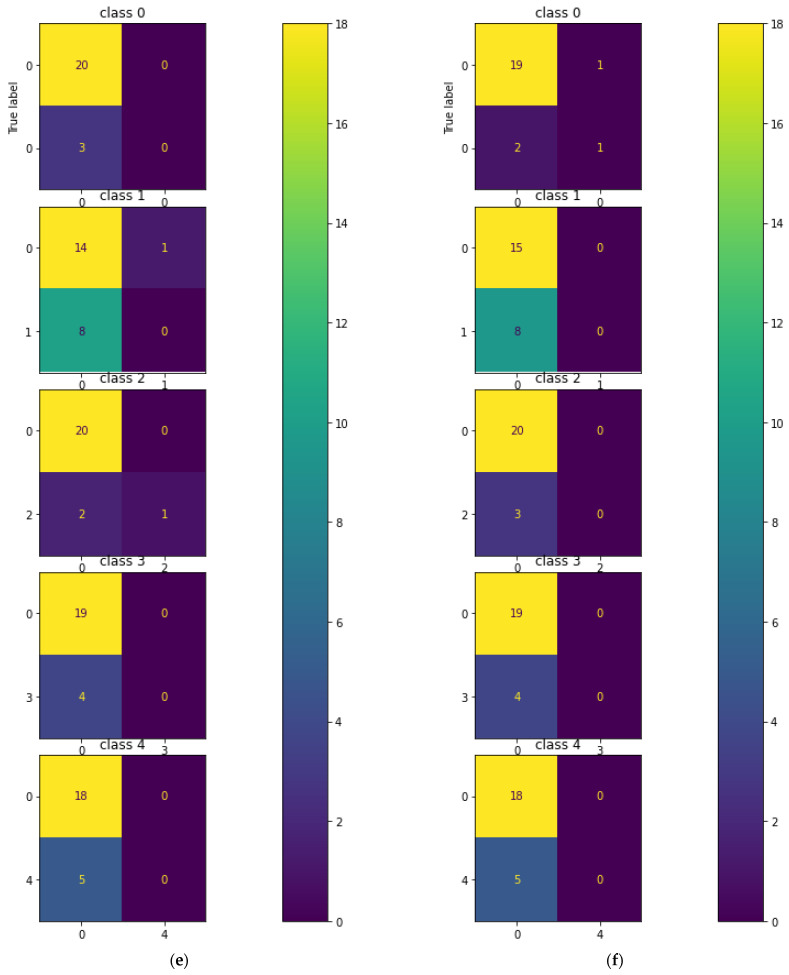
Five-class error matrix for the phase-only image dataset: (**a**) CNN; (**b**) KNN; (**c**) MLP; (**d**) DT; (**e**) RF; (**f**) ET.

Figure 15 shows that the validation error was higher compared to the training error and that the accuracy of the training set was higher compared to that of the validation set. This showed that the CNN model was overfitting. The testing of the CNN for the phase-only image dataset on the test set was performed in the same manner as that of the hologram dataset. The confusion matrix obtained by the CNN for the five classes is shown in Figure 16.

Further, the performance of the five-class classification task was described by the machine learning classifiers. The testing of the machine learning classifiers for the phase-only image dataset on the test set was performed in the same manner as that of the hologram dataset. Figure 16 also describes the confusion matrix for all five classes obtained by the KNN, MLP, DT, RF, and ET classifiers for the phase-only image dataset. The performance metrics obtained by the CNN, KNN, MLP, DT, RF, and ET classifiers on the phase-only image dataset are shown in Table 6.

Table 6 shows that the CNN and RF classifiers had a greater accuracy for Class-e and Class-c compared to the other classes. The KNN and DT classifiers had a larger accuracy for Class-d compared to the other classes. The MLP classifier had a higher accuracy for Class-b compared to the other classes. The ET classifier achieved a higher accuracy for Class-a and Class-c compared to the other classes. Table 7 shows the computational costs and complexity parameters such as the floating-point operations (FLOPs), training time, and test time for the CNN and the other machine learning classifiers (KNN, MLP, DT, RF, and ET).

**Figure 17:**
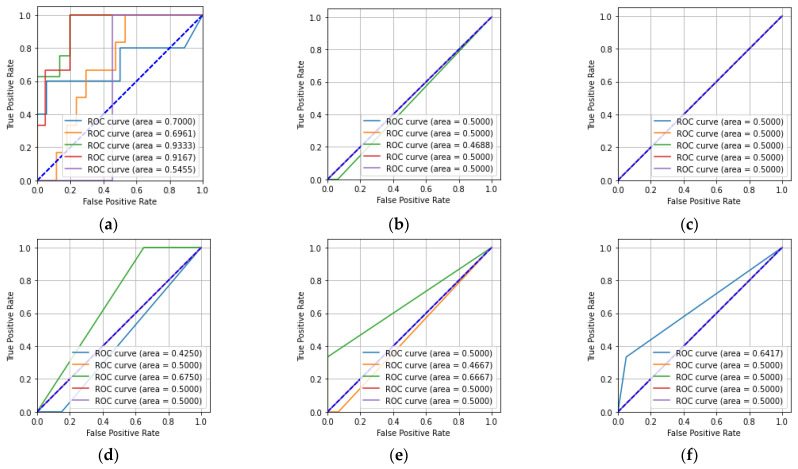
ROCs for the phase-only image dataset: (**a**) CNN; (**b**) KNN; (**c**) MLP; (**d**) DT; (**e**) RF; (**f**) ET.

In Table 7, it can be seen that the number of floating-point operations (FLOPs) for the CNN was higher compared to those of the machine learning classifiers. In Table 7, it also can be seen that the training time and test time for the CNN were greater compared to the machine learning classifiers. Figure 17 depicts the ROCs obtained from the CNN, KNN, MLP, DT, RF, and ET classifiers for the phase-only image dataset.

**Figure 18:**
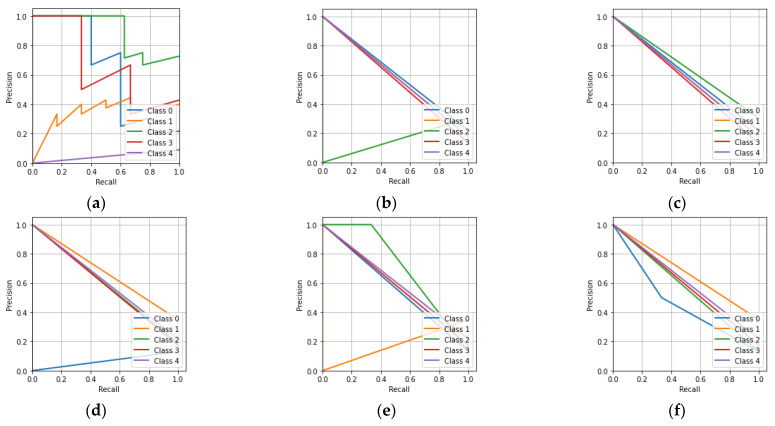
Precision-recall characteristics for the phase-only image dataset: (**a**) CNN; (**b**) KNN; (**c**) MLP; (**d**) DT; (**e**) RF; (**f**) ET.

In Figure 17a, it can be seen that the CNN achieved the highest AUC value of 0.93 for Class-c compared to the other classes. The remaining machine learning classifiers achieved a lower AUC value for Class-c compared to that of the CNN. Overall, the CNN had better AUC values for all of the classes compared to the KNN, MLP, DT, RF, and ET classifiers. The precision-recall characteristics for all five classes obtained by the CNN, KNN, MLP, DT, RF, and ET classifiers for the phase-only image dataset are shown in Figure 18.

Figure 18a shows that the CNN had a lower precision when the recall approached unity for all the classes. Similarly, for the remaining precision-recall characteristics, it can be seen that the KNN, MLP, DT, RF, and ET classifiers also had a lower precision when the recall approached unity for all the classes.

### 5.4. Training of CNN for Multi-Output Regression on Holograms

**Figure 19:**
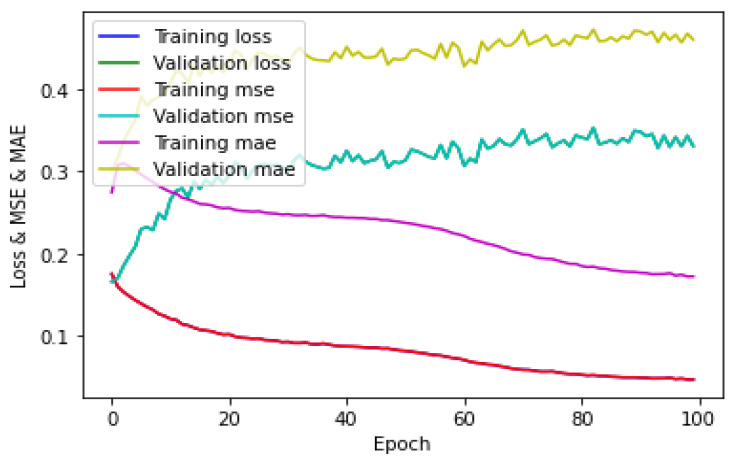
Loss/MSE/MAE plot for hologram dataset.

The training of the CNN for the five-class regression on the hologram dataset was performed in the same manner as that of the five-class classification on the hologram dataset. Figure 19 shows the loss/MSE/MAE plot obtained by the CNN for the training/validation sets; it can be seen that the validation error was higher than the training error. The loss and MSE plots were the same for both training/validation sets. Further, Figure 19 shows that the validation MAE was greater than the training MAE. This showed that the CNN was not correctly fitting. The testing of the CNN was performed separately on a batch size of 23 images for the test set on the hologram dataset. The evaluation metrics such as the mean absolute error (MAE), R^2^ score (coefficient of determination), and explained-variance (EV) regression score were used to measure the performance of the five-class regression task. The evaluation metrics obtained by the CNN are shown in Table 8. Further, the evaluation metrics obtained by the CNN were compared with those of the machine learning regressors. The evaluation metrics obtained by the KNN, MLP, DT, RF, and ET regressors with a batch size of 23 images for the hologram dataset on the test set are also shown in Table 8.

Table 8 shows that the MLP regressor had a better performance for the five-class regression tasks on the test set with a stable EV regression score of 0.00 compared to the CNN and other regressors. The testing of the CNN for the validation set on the hologram dataset was also performed with a batch size of 20 images. The evaluation metrics obtained by the CNN and the other machine learning regressors for the validation set are shown in Table 9. The machine learning regressors were also tested on the validation set with a batch size of 20 images for the hologram dataset.

Table 9 shows that the MLP regressor had a consistent performance on the validation set compared to the CNN and the other machine learning regressors with a fixed EV regression score of 0.00.

### 5.5. Training of CNN for Multi-Output Regression on Concatenated Intensity–Phase Image Dataset

**Figure 20:**
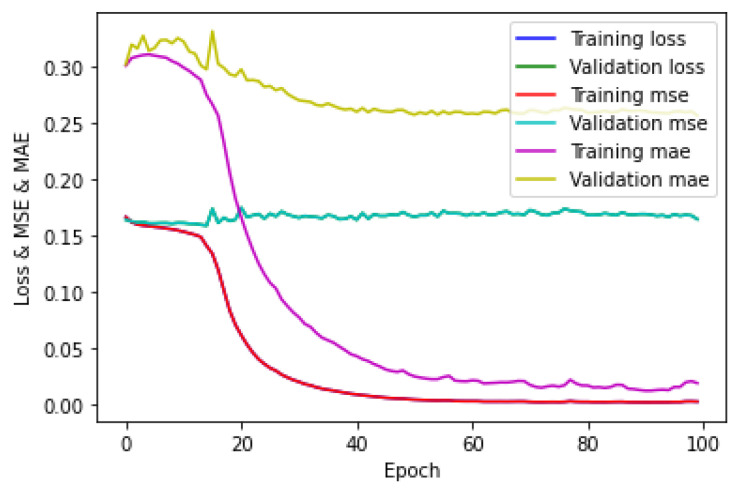
Loss/MSE/MAE plot on training/validation sets for whole information dataset.

The training of the CNN for the five-class regression on the concatenated intensity–phase image dataset was performed in the same manner as that of the five-class classification on the hologram dataset. Figure 20 shows the loss/MSE/MAE plot obtained by the CNN on the training/validation sets.

Figure 20 shows that the validation error was greater than the training error. The loss and MSE plots were the same for both the training/validation sets. Further, it can be seen that the validation MAE was greater than the training MAE. Therefore, it can be said that the CNN model was not correctly fitting. The testing of the CNN for the concatenated intensity–phase image dataset on the test set was performed in the same manner as that of the hologram dataset. The evaluation metrics obtained by the CNN are shown in Table 10. Further, the evaluation metrics obtained by the CNN were compared with certain machine learning regressors. The evaluation metrics obtained by those machine learning regressors on the test set are also shown in Table 10. The testing of the machine learning regressors for the concatenated intensity-phase image dataset on the test set was performed in the same manner as that of the hologram dataset.

Table 10 shows that the MLP regressor had a better performance for the five-class regression tasks on the test set with a fixed EV regression score of 0.00 compared to the CNN and the other machine learning regressors. The testing of the CNN for the concatenated intensity–phase image dataset on the validation set was performed in the same manner as that of the hologram dataset. The evaluation metrics obtained by the CNN and the other machine learning regressors on the validation set are shown in Table 11. The testing of the machine learning regressors for the whole information dataset on the validation set was performed in the same manner as that of the hologram dataset.

Table 11 shows that the RF regressor had a better performance on the validation set compared to the CNN and the other machine learning regressors with a stable EV regression score of 0.01.

### 5.6. Training of CNN for Multi-Output Regression on Phase-Only Information

**Figure 21:**
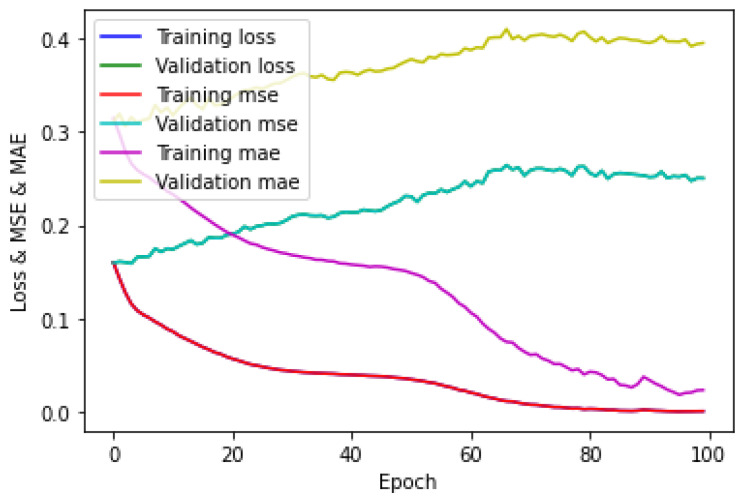
Loss/MSE/MAE plot for training/validation sets of the phase-only image dataset.

The training of the CNN for the five-class regression on the phase-only image dataset was performed in the same manner as that of the five-class classification on the hologram dataset. The loss/MSE/MAE plot for the training/validation sets is provided in Figure 21, which shows that the error for the training set was lower than the error for the validation set. The loss and MSE plots were the same as those depicted in Figure 21. Further, it can also be seen in Figure 21 that the validation MAE was higher compared to the training MAE. This showed that the CNN model was overfitting. The testing of the CNN for the phase-only image dataset on the test set was performed in the same manner as that of the hologram dataset. Next, these evaluation metrics obtained by the CNN on the test set were compared with certain machine learning regressors. The evaluation metrics obtained from the CNN and the machine learning regressors on the test set are shown in Table 12. The testing of machine learning regressors for phase-only image dataset on the test set was performed in the same manner as that of the hologram dataset.

Table 12 shows that the MLP regressor had a better performance for the five-class regression tasks on the test set with a fixed EV regression score of 0.00 compared to the CNN, KNN, DT, RF, and ET regressors. The testing of the CNN for the phase-only image dataset on the validation set was performed in the same manner as that of the hologram dataset. The evaluation metrics obtained by the CNN and the other machine learning regressors on the validation set are shown in Table 13. The testing of the machine learning regressors for the validation set on the phase-only image dataset was performed in the same manner as that of the hologram dataset.

Table 13 shows that the MLP regressor had a good performance on the validation set compared to the CNN and the other machine learning regressors with a stable EV regression score of 0.00.

## 6. Conclusions

In this paper, digital holographic information in datasets comprising holograms, reconstructed intensity and phase images combined to form concatenated intensity–phase images, and phase-only images was used for the proposed multi-class classification and multi-output regression tasks by using deep learning and machine learning techniques. Each dataset comprised 2268 images separately to perform the multi-class classification and multi-output regression tasks. A deep CNN was used on all three datasets independently to perform the five-class classification and regression tasks. The five-class classification and regression tasks in supervised learning applied to the digital holographic information of eighteen 3D objects using deep CNN was equivalent to 3D object allocation and prediction performed on the digital holographic datasets that produced discrete and continuous labels as output, which justified the rationale behind the present work. For the five-class classification task, the results such as the error/accuracy plots and error matrix, evaluation metrics, receiver operating characteristics (ROCs), and precision-recall characteristics were shown for the confirmation of the work. Similarly, for the five-class regression task, the results such as the error/mean square error (MSE)/mean absolute error (MAE) plots and evaluation metrics were shown for the confirmation of the work. The CNN overfitted all three datasets as shown by the error/accuracy graphs. The ML classifiers had better AUC values for different classes on the datasets of holograms and concatenated intensity–phase images when compared to the CNN. Further, the CNN was found to have a higher AUC value for all five classes on the phase-only image dataset when compared to the other machine learning classifiers. Similarly, the CNN overfitted all three datasets as obtained in the loss/MSE/MAE curves on the training/validation sets. Further, the MLP regressor had a better performance on the test/validation sets for the hologram and phase-only image datasets with a fixed EV regression score of 0.00 compared to the CNN and the other machine learning regressors [51]. The RF regressor had a better performance on the validation set for the concatenated intensity–phase image dataset with a stable EV regression score of 0.01 compared to the CNN and the other regressors [52]. Therefore, we concluded that both the CNN and the machine learning classifiers and regressors (KNN, MLP, DT, RF, and ET) had a superior performance in both the five-class classification and regression tasks for all three datasets.

## Figures and Tables

**Table 1:** Model Summary of CNN.

Layer	Input	Output	Number of Parameters
Conv1	160 × 160 × 8	158 × 158 × 8	224
MaxPooling2D	158 × 158 × 8	79 × 79 × 8	0
Conv2	79 × 79 × 16	77 × 77 × 16	1168
MaxPooling2D	77 × 77 × 16	38 × 38 × 16	0
Conv3	38 × 38 × 32	36 × 36 × 32	4640
MaxPooling2D	36 × 36 × 32	18 × 18 × 32	0
Conv4	18 × 18 × 64	16 × 16 × 64	18,496
MaxPooling2D	16 × 16 × 64	8 × 8 × 64	0
Fully connected	4096	16	65,552
Output	16	5	85
Total number of parameters			90,165

**Table 2:** Evaluation metrics for the CNN, KNN, MLP, DT, RF, and ET classifiers on the hologram dataset.

**Class and Metric**	**Class-a**	**Class-b**	**Class-c**	**Class-d**	**Class-e**	**Micro Average**	**Macro Average**	**Weighted Average**	**Samples Average**	**CNN**
Accuracy	0.78	0.65	0.61	0.74	0.48					
Precision	0.00	0.00	0.38	0.00	0.00	0.13	0.07	0.11	0.13	
Recall	0.00	0.00	0.43	0.00	0.00	0.13	0.09	0.13	0.13	
F1-Score	0.00	0.00	0.40	0.00	0.00	0.13	0.08	0.12	0.13	
**Class and Metric**	**Class-a**	**Class-b**	**Class-c**	**Class-d**	**Class-e**	**Micro Average**	**Macro Average**	**Weighted Average**	**Samples Average**	**KNN**
Accuracy	0.87	0.91	0.57	0.57	0.83					
Precision	0.00	0.00	0.00	0.22	0.00	0.20	0.04	0.05	0.09	
Recall	0.00	0.00	0.00	0.40	0.00	0.09	0.08	0.09	0.09	
F1-Score	0.00	0.00	0.00	0.29	0.00	0.12	0.06	0.06	0.09	
**Class and Metric**	**Class-a**	**Class-b**	**Class-c**	**Class-d**	**Class-e**	**Micro Average**	**Macro Average**	**Weighted Average**	**Samples Average**	**MLP**
Accuracy	0.13	0.91	0.43	0.22	0.13					
Precision	0.13	0.00	0.43	0.22	0.13	0.23	0.18	0.27	0.23	
Recall	1.00	0.00	1.00	1.00	1.00	0.91	0.80	0.91	0.91	
F1-Score	0.23	0.00	0.61	0.36	0.23	0.37	0.28	0.40	0.37	
**Class and Metric**	**Class-a**	**Class-b**	**Class-c**	**Class-d**	**Class-e**	**Micro Average**	**Macro Average**	**Weighted Average**	**Samples Average**	**DT**
Accuracy	0.65	0.87	0.48	0.39	0.87					
Precision	0.00	0.00	0.00	0.20	0.00	0.13	0.04	0.04	0.13	
Recall	0.00	0.00	0.00	0.60	0.00	0.13	0.12	0.13	0.13	
F1-Score	0.00	0.00	0.00	0.30	0.00	0.13	0.06	0.07	0.13	
**Class and Metric**	**Class-a**	**Class-b**	**Class-c**	**Class-d**	**Class-e**	**Micro Average**	**Macro Average**	**Weighted Average**	**Samples Average**	**RF**
Accuracy	0.65	0.87	0.83	0.65	0.87					
Precision	0.00	0.00	0.00	0.00	0.00	0.00	0.00	0.00	0.00	
Recall	0.00	0.00	0.00	0.00	0.00	0.00	0.00	0.00	0.00	
F1-Score	0.00	0.00	0.00	0.00	0.00	0.00	0.00	0.00	0.00	
**Class and Metric**	**Class-a**	**Class-b**	**Class-c**	**Class-d**	**Class-e**	**Micro Average**	**Macro Average**	**Weighted Average**	**Samples Average**	**ET**
Accuracy	0.65	0.87	0.83	0.61	0.87					
Precision	0.00	0.00	0.00	0.00	0.00	0.00	0.00	0.00	0.00	
Recall	0.00	0.00	0.00	0.00	0.00	0.00	0.00	0.00	0.00	
F1-Score	0.00	0.00	0.00	0.00	0.00	0.00	0.00	0.00	0.00	

**Table 3:** Computational costs and complexities for the CNN, KNN, MLP, DT, RF, and ET classifiers on the hologram dataset.

Parameter	CNN	KNN	MLP	DT	RF	ET
Floating-point operations (FLOPs)	45,223,104	384,000	76,805	76,807	1,536,000	1,536,000
Training time (s)	6560	175	190	125	116	119
Test time (s)	163	94	80	67	71	78

**Table 4:** Evaluation metrics for the CNN, KNN, MLP, DT, RF, and ET classifiers on the whole information dataset.

**Class and Metric**	**Class-a**	**Class-b**	**Class-c**	**Class-d**	**Class-e**	**Micro Average**	**Macro Average**	**Weighted Average**	**Samples Average**	**CNN**
Accuracy	0.91	0.61	0.74	0.74	0.61					
Precision	0.00	0.50	0.38	0.00	0.27	0.30	0.23	0.31	0.30	
Recall	0.00	0.11	0.75	0.00	0.75	0.30	0.32	0.30	0.30	
F1-Score	0.00	0.18	0.50	0.00	0.40	0.30	0.22	0.23	0.30	
**Class and Metric**	**Class-a**	**Class-b**	**Class-c**	**Class-d**	**Class-e**	**Micro Average**	**Macro Average**	**Weighted Average**	**Samples Average**	**KNN**
Accuracy	0.74	0.83	0.91	0.61	0.83					
Precision	0.60	0.00	0.00	0.00	0.00	0.38	0.12	0.18	0.13	
Recall	0.43	0.00	0.00	0.00	0.00	0.13	0.09	0.13	0.13	
F1-Score	0.50	0.00	0.00	0.00	0.00	0.19	0.10	0.15	0.13	
**Class and Metric**	**Class-a**	**Class-b**	**Class-c**	**Class-d**	**Class-e**	**Micro Average**	**Macro Average**	**Weighted Average**	**Samples Average**	**MLP**
Accuracy	0.30	0.83	0.09	0.26	0.17					
Precision	0.30	0.00	0.09	0.26	0.17	0.21	0.17	0.20	0.21	
Recall	1.00	1.00	1.00	1.00	1.00	0.83	0.80	0.83	0.83	
F1-Score	0.47	0.00	0.16	0.41	0.30	0.33	0.27	0.32	0.33	
**Class and Metric**	**Class-a**	**Class-b**	**Class-c**	**Class-d**	**Class-e**	**Micro Average**	**Macro Average**	**Weighted Average**	**Samples Average**	**DT**
Accuracy	0.91	0.78	0.74	0.57	0.83					
Precision	0.00	0.60	0.00	0.27	0.00	0.38	0.17	0.22	0.26	
Recall	0.00	0.50	0.00	0.60	0.00	0.26	0.22	0.26	0.26	
F1-Score	0.00	0.55	0.00	0.37	0.00	0.31	0.18	0.22	0.26	
**Class and Metric**	**Class-a**	**Class-b**	**Class-c**	**Class-d**	**Class-e**	**Micro Average**	**Macro Average**	**Weighted Average**	**Samples Average**	**RF**
Accuracy	0.91	0.74	0.70	0.78	0.87					
Precision	0.00	0.00	0.33	0.00	1.00	0.50	0.27	0.26	0.09	
Recall	0.00	0.00	0.17	0.00	0.25	0.09	0.08	0.09	0.09	
F1-Score	0.00	0.00	0.22	0.00	0.40	0.15	0.12	0.13	0.09	
**Class and Metric**	**Class-a**	**Class-b**	**Class-c**	**Class-d**	**Class-e**	**Micro Average**	**Macro Average**	**Weighted Average**	**Samples Average**	**ET**
Accuracy	0.91	0.74	0.78	0.70	0.83					
Precision	0.00	0.00	1.00	0.00	0.00	0.33	0.20	0.26	0.04	
Recall	0.00	0.00	0.17	0.00	0.00	0.04	0.03	0.04	0.04	
F1-Score	0.00	0.00	0.29	0.00	0.00	0.08	0.06	0.07	0.04	

**Table 5:** Computational costs and complexities for the CNN, KNN, MLP, DT, RF, and ET classifiers on the whole information dataset.

Parameter	CNN	KNN	MLP	DT	RF	ET
Floating-point perations (FLOPs)	4,522,3104	384,000	76,805	76,807	1,536,000	1,536,000
Training time(s)	5012	164	178	134	121	131
Test time(s)	139	89	73	62	64	72

**Table 6:** Evaluation metrics for the CNN, KNN, MLP, DT, RF, and ET classifiers on the phase-only image dataset.

**Class and Metric**	**Class-a**	**Class-b**	**Class-c**	**Class-d**	**Class-e**	**Micro Average**	**Macro Average**	**Weighted Average**	**Samples Average**	**CNN**
Accuracy	0.78	0.65	0.65	0.87	0.91					
Precision	0.00	0.00	0.50	0.50	0.00	0.43	0.20	0.24	0.43	
Recall	0.00	0.00	1.00	0.67	0.00	0.43	0.33	0.43	0.43	
F1-Score	0.00	0.00	0.67	0.57	0.00	0.43	0.25	0.31	0.43	
**Class and Metric**	**Class-a**	**Class-b**	**Class-c**	**Class-d**	**Class-e**	**Micro Average**	**Macro Average**	**Weighted Average**	**Samples Average**	**KNN**
Accuracy	0.78	0.83	0.65	0.87	0.83					
Precision	0.00	0.00	0.00	0.00	0.00	0.00	0.00	0.00	0.00	
Recall	0.00	0.00	0.00	0.00	0.00	0.00	0.00	0.00	0.00	
F1-Score	0.00	0.00	0.00	0.00	0.00	0.00	0.00	0.00	0.00	
**Class and Metric**	**Class-a**	**Class-b**	**Class-c**	**Class-d**	**Class-e**	**Micro Average**	**Macro Average**	**Weighted Average**	**Samples Average**	**MLP**
Accuracy	0.22	0.83	0.30	0.13	0.17					
Precision	0.22	0.00	0.30	0.13	0.17	0.21	0.17	0.19	0.21	
Recall	1.00	0.00	1.00	1.00	1.00	0.83	0.80	0.83	0.83	
F1-Score	0.36	0.00	0.47	0.23	0.30	0.33	0.27	0.30	0.33	
**Class and Metric**	**Class-a**	**Class-b**	**Class-c**	**Class-d**	**Class-e**	**Micro Average**	**Macro Average**	**Weighted Average**	**Samples Average**	**DT**
Accuracy	0.74	0.65	0.43	0.83	0.78					
Precision	0.00	0.00	0.19	0.00	0.00	0.16	0.04	0.02	0.13	
Recall	0.00	0.00	1.00	0.00	0.00	0.13	0.20	0.13	0.13	
F1-Score	0.00	0.00	0.32	0.00	0.00	0.14	0.06	0.04	0.13	
**Class and Metric**	**Class-a**	**Class-b**	**Class-c**	**Class-d**	**Class-e**	**Micro Average**	**Macro Average**	**Weighted Average**	**Samples Average**	**RF**
Accuracy	0.87	0.61	0.91	0.83	0.78					
Precision	0.00	0.00	1.00	0.00	0.00	0.50	0.20	0.13	0.04	
Recall	0.00	0.00	0.33	0.00	0.00	0.04	0.07	0.04	0.04	
F1-Score	0.00	0.00	0.50	0.00	0.00	0.08	0.10	0.07	0.04	
**Class and Metric**	**Class-a**	**Class-b**	**Class-c**	**Class-d**	**Class-e**	**Micro Average**	**Macro Average**	**Weighted Average**	**Samples Average**	**ET**
Accuracy	0.87	0.65	0.87	0.83	0.78					
Precision	0.50	0.00	0.00	0.00	0.00	0.50	0.10	0.07	0.04	
Recall	0.33	0.00	0.00	0.00	0.00	0.04	0.07	0.04	0.04	
F1-Score	0.40	0.00	0.00	0.00	0.00	0.08	0.08	0.05	0.04	

**Table 7:** Computational costs and complexities for the CNN, KNN, MLP, DT, RF, and ET classifiers on the phase-only image dataset.

Parameter	CNN	KNN	MLP	DT	RF	ET
Floating-point operations (FLOPs)	45,223,104	384,000	76,805	76,807	1,536,000	1,536,000
Training time(s)	5635	159	181	141	123	134
Test time(s)	96	79	68	61	58	67

**Table 8:** Evaluation metrics for test set of the hologram dataset.

Metric	CNN	KNN	MLP	DT	RF	ET
MAE	0.46	0.30	0.48	0.34	0.35	0.32
R^2^ score	−1.20	−0.09	−1.13	−0.74	−0.40	−0.26
EV regression score	−0.83	−0.01	0.00	-0.64	−0.27	−0.13

**Table 9:** Evaluation metrics for validation set of the hologram dataset.

Metric	CNN	KNN	MLP	DT	RF	ET
MAE	0.46	0.34	0.42	0.31	0.31	0.32
R^2^ score	−1.35	−0.65	−1.10	−0.44	−0.14	−0.27
EV regression score	−0.77	−0.33	0.00	−0.33	−0.13	−0.19

**Table 10:** Evaluation metrics for test set of the whole information dataset.

Metric	CNN	KNN	MLP	DT	RF	ET
MAE	0.33	0.28	0.47	0.33	0.31	0.32
R^2^ score	−0.93	−0.11	−1.26	−1.01	−0.14	−0.61
EV regression score	−0.30	−0.02	0.00	−0.79	−0.02	−0.42

**Table 11:** Evaluation metrics for validation set on whole information dataset.

Metric	CNN	KNN	MLP	DT	RF	ET
MAE	0.25	0.31	0.51	0.28	0.31	0.28
R^2^ score	−0.17	−0.36	−2.01	−0.45	−0.13	−0.23
EV regression score	−0.16	−0.23	0.00	−0.43	0.01	−0.06

**Table 12:** Evaluation metrics for test set on phase-only image dataset.

Metric	CNN	KNN	MLP	DT	RF	ET
MAE	0.36	0.33	0.44	0.30	0.32	0.30
R^2^ score	−0.58	−0.38	−0.86	−0.70	−0.17	−0.19
EV regression score	−0.20	−0.10	0.00	−0.62	−0.10	−0.13

**Table 13:** Evaluation metrics for validation set of phase-only image dataset.

Metric	CNN	KNN	MLP	DT	RF	ET
MAE	0.44	0.35	0.44	0.37	0.34	0.33
R^2^ score	−0.79	−0.43	−0.88	−1.51	−0.41	−0.56
EV regression score	−0.62	−0.23	0.00	−1.21	−0.15	−0.30

## Data Availability

Not applicable.

## Supplementary Materials

The following supporting information can be downloaded at: https://www.mdpi.com/article/10.3390/s23031095/s1, Figure S1: 3D object used in the off-axis digital holographic recording geometry; Figure S2: Off-axis digital holographic recording geometry used for the recording of hologram of 3D object; Figure S3: General Multi-Class Confusion Matrix.
