# Supplementary material for: Multi-Class Classification and Multi-Output Regression of Three-Dimensional Objects Using Artificial Intelligence Applied to Digital Holographic Information
Source: Sensors (Basel). 2023 Jan 17;23(3):1095. doi: 10.3390/s23031095 (PMC9920031; doi:10.3390/s23031095)
Supplement: Supplementary file 1 [file sensors-23-01095-s001.zip › sensors-2078115-supplementary.pdf]

sensors

Supplementary Materials

Multi-class classification and Multi-output regression of three-dimensional objects using artificial intelligence applied to digital holographic information

Uma Mahesh R N and Anith Nelleri *

School of Electronics Engineering, Vellore Institute of Technology (VIT), Chennai, Tamilnadu, India – 600127.

*Corresponding author: *anith.nelleri@vit.ac.in*

Abstract

This document contains the Supplementary Materials for "Multi-class classification and Multi-output regression of three dimensional objects using artificial intelligence applied to digital holographic information". This material describes the digital hologram sensing, retrieval, and processing of 3D information using deep learning and machine learning techniques. This material also contains the mathematical model of the deep convolutional neural network (CNN) that was used for the above problem and the general multi-class confusion matrix that was used for comparison of the deep CNN with machine learning algorithms for the above problem at hand.

Section S1

Section S1.1. Mathematical modeling and construction of 3D objects used in off-axis digital Fresnel holography

In this section, the construction and mathematical modeling of the 3D objects used in the off-axis digital holographic recording geometry is described. The schematic of one of the 3D objects is shown in Figure S1.

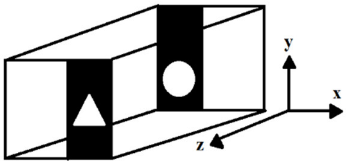

Figure S1. The 3D circle–triangle object used in the off-axis digital holographic recording geometry. Circle: 2 mm in diameter; triangle: 2 mm in x and y directions. The distance between the first plane and second plane was 8 mm in the z direction.

Figure S1 shows the schematic of one of the 3D objects with the features of a circle in the first plane and a triangle in the second plane separated by a distance of $z = 8\,mm$. The schematics of the remaining seventeen 3D objects were similar to the one shown in Figure S1 but with different features of the first and second planes. A 3D object was characterized by its intensity and phase information. Let the amplitude and phase information in the first plane be

$A_1(x, y)exp\ (i\phi_1(x, y))$. Similarly, let the amplitude and phase information in the second plane be $A_2(x, y)exp\ (i\phi_2(x, y))$. When light passed through the first plane, the amplitude and phase information of the object with the features of the first plane were obtained. Then, after propagating by a distance of z using free space propagation, the amplitude and phase information of the next object features in the second plane were also obtained. Next, **t**he total distribution immediately after the second plane is given by Eqn. (S1):

$$A_u(x, y) = \left\{A_1(x, y)\exp\left(i\phi_1(x, y)\right)\otimes \exp\left(\frac{i\pi}{\lambda z}(x^2 + y^2)\right)\right\} A_2(x, y)\exp\left(i\phi_2(x, y)\right) \quad \text{(S1)}$$

where '⊗' represents the convolution operator; and the terms $A_1(x, y)$, $\phi_1(x, y)$ and $A_2(x, y)$, $\phi_2(x, y)$ represent the 3D information in the form of the amplitude and phase of both the first and second planes, respectively. By using off-axis digital Fresnel holography, the single digital hologram was formed by the interference between the object wave ($A_u(.)$) and plane reference wave $R(.) = \exp\ (i\theta)$ at an angle θ. Now, the complete complex field distribution of the 3D object function $A_u(x, y)$ positioned at a distance d from the recording plane is represented by Eqn. (S2):

$$U(x', y', d) = \frac{e^{ik_1 d}}{i\lambda d} \iint A_u(x, y, 0) exp \frac{i\pi}{\lambda d}[(x' - x)^2 + (y' - y)^2] dx\, dy \quad \text{(S2)}$$

where $\mathrm{U}(.)$ represents the complete complex Fresnel field distribution at the recording plane of the 3D object; and (x, y), (x', y') denote the object plane and recording plane coordinates, respectively. The propagation of the 3D information of the object was conducted by using a laser source with a wavelength of λ. The recorded off-axis digital Fresnel hologram $H(x', y')$ is given by Eqn. (S3):

$$H(x', y') = |U(x', y') + R(x', y')|^2 = |O_u exp\ (i\vec{k}_1.\vec{r}) + exp\ (i\vec{k}_2.\vec{r})|^2 \quad \text{(S3)}$$

where $\vec{k}_1$ and $\vec{k}_2$ are the propagation wave vectors of the object and reference waves, respectively. The resultant wave vector $\vec{K}$ of the interference fringe pattern is given by Eqn. (S4):

$$|\vec{K}| = |\vec{k}_1 - \vec{k}_2| = \frac{4\pi}{\lambda} sin\left(\frac{\theta}{2}\right) \quad \text{(S4)}$$

where θ represents the angle between the two interfering waves [1].

Section S1.2. Off-axis digital holographic recording geometry and numerical reconstruction for obtaining the 3D information of objects

Digital holography is a 3-D imaging technique that is used for the recording of a hologram of a 3D object. Figure S2 shows a schematic of the off-axis digital holographic recording geometry.

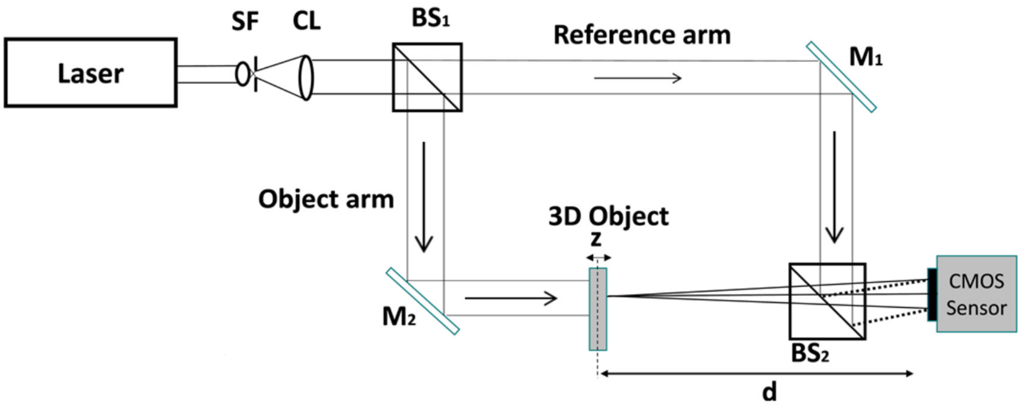

Figure S2. Off-axis digital holographic recording geometry used for the recording of the holograms of 3D objects. SF: spatial filter assembly; CL: collimation lens; BS: beam splitter; M: mirror; CMOS: camera sensor.

The light beam emitted from the He-Ne laser source with a wavelength λ = 632.8 nm was passed through the spatial filter to remove the unwanted interference patterns in the laser beam. The filtered laser beam was collimated through the collimation lens. The collimated beam was separated through the beam splitter (BS_1) into two arms; i.e., the object arm and the reference arm. In the object arm, the 3D object was placed at a distance 'd' from the CMOS sensor. The light beam scattered from the 3D object formed the object wave and another arm formed the reference wave. At the exit of (BS_2), the object wave and reference wave interfered at an angle θ to form the off-axis digital Fresnel hologram. The CMOS sensor was used for the recording of the off-axis digital Fresnel hologram [1]. After the hologram was recorded, the complex-wave retrieval method [2] was applied on the recorded hologram of the 3D object to obtain the complex wave field of the object at the recording plane. Further, an inverse Fresnel transform was applied on the retrieved complex wave field to obtain the 2D digital complex-valued image that contained the 3D information of the object. Finally, datasets for the sensed holograms, the images with a retrieved intensity and phase extracted from the 2D digital complex-valued image combined in a concatenated manner, and the phase-only images were used for the five-class classification and regression tasks of the 3D objects by using deep CNN and machine learning algorithms.

Section S1.3. 3D object set formation of datasets of sensed holograms, concatenated intensity–phase images, and phase-only images

The set of 18 different 3D objects that were considered in this study were named based on their features in the first and second planes: *circle–pentagon* (a_{d_i}), *circle–triangle* (b_{d_i}), *circle–square* (c_{d_i}), *circle–rectangle* (e_{d_i}), *square–circle* (f_{d_i}), *square–triangle* (g_{d_i}), *square–rectangle* (h_{d_i}), *square–pentagon* (k_{d_i}), *triangle–circle* (l_{d_i}), *triangle–square* (m_{d_i}), *triangle–*

rectangle (n_{d_i}), *triangle–pentagon* (o_{d_i}), *pentagon–circle* (p_{d_i}), *pentagon–square* (q_{d_i}), *pentagon–triangle* (r_{d_i}), *rectangle–circle* (s_{d_i}), *rectangle–square* (t_{d_i}), and *rectangle–triangle* (u_{d_i}), which constituted the $'\{T\}'$ [3]:

$$\{T\} \in \{a_{d_i}, b_{d_i}, c_{d_i}, e_{d_i}, f_{d_i}, g_{d_i}, h_{d_i}, k_{d_i}, l_{d_i}, m_{d_i}, n_{d_i}, o_{d_i}, p_{d_i}, q_{d_i}, r_{d_i}, s_{d_i}, t_{d_i}, u_{d_i}\},$$
$$d_i \in \{d_1, d_2, d_3, \dots\dots\dots d_i \dots\dots., d_N\} \quad \text{(S5)}$$

where $'d_i'$ represents the distance between the recording plane and object plane and i denotes the indices of the individual objects. The objects were positioned at different distances in the 3D scene. The 3D objects shown in $'\{T\}'$ represented the real-valued digital holograms; the computationally retrieved 2D digital complex-valued images of the 3D objects are presented in $'\{RT\}'$ [3]:

$$\{RT\} \in \{Ra_{d_i}, Rb_{d_i}, Rc_{d_i}, Re_{d_i}, Rf_{d_i}, Rg_{d_i}, Rh_{d_i}, Rk_{d_i}, Rl_{d_i}, Rm_{d_i}, Rn_{d_i}, Ro_{d_i},$$
$$Rp_{d_i}, Rq_{d_i}, Rr_{d_i}, Rs_{d_i}, Rt_{d_i}, Ru_{d_i}\},\ d_i \in \{d_1, d_2, d_3, \dots\dots\dots d_i \dots\dots., d_N\} \quad \text{(S6)}$$

The $'\{RT\}'$ was further separated into $'\{RT_{IN}\}'$ and $'\{RT_{PH}\}'$ as shown in Eqns. (S7) and (S8) respectively. The $'\{RT_{IN}\}'$ was formed by taking the absolute of $'\{RT\}'$ [3]:

$$\{RT_{IN}\} \in \{Ra_{d_{i,IN}}, Rb_{d_{i,IN}}, Rc_{d_{i,IN}}, Re_{d_{i,IN}}, Rf_{d_{i,IN}}, Rg_{d_{i,IN}}, Rh_{d_{i,IN}},$$
$$Rk_{d_{i,IN}}, Rl_{d_{i,IN}}, Rm_{d_{i,IN}}, Rn_{d_{i,IN}}, Ro_{d_{i,IN}}, Rp_{d_{i,IN}}, Rq_{d_{i,IN}}, Rr_{d_{i,IN}}, Rs_{d_{i,IN}}, Rt_{d_{i,IN}}, Ru_{d_{i,IN}}\},$$
$$d_i \in \{d_1, d_2, d_3, \dots\dots\dots d_i \dots\dots., d_N\} \quad \text{(S7)}$$

where index IN denotes the set comprising the intensity images. Similarly, the $'\{RT_{PH}\}'$ was formed by taking the angle of $'\{RT\}'$ [3]:

$$\{RT_{PH}\} \in \{Ra_{d_{i,PH}}, Rb_{d_{i,PH}}, Rc_{d_{i,PH}}, Re_{d_{i,PH}}, Rf_{d_{i,PH}}, Rg_{d_{i,PH}}, Rh_{d_{i,PH}},$$
$$Rk_{d_{i,PH}}, Rl_{d_{i,PH}}, Rm_{d_{i,PH}}, Rn_{d_{i,PH}}, Ro_{d_{i,PH}}, Rp_{d_{i,PH}}, Rq_{d_{i,PH}}, Rr_{d_{i,PH}}, Rs_{d_{i,PH}}, Rt_{d_{i,PH}}, Ru_{d_{i,PH}}\},$$
$$d_i \in \{d_1, d_2, d_3, \dots\dots\dots d_i \dots\dots., d_N\} \quad \text{(S8)}$$

where index PH denotes the set comprising the phase images. The $'\{RT_{IN}\}'$ and $'\{RT_{PH}\}'$ were further combined via the method of concatenation to form the concatenated intensity–phase image dataset $'\{RT_{INPH}\}'$ [3]:

$$\{RT_{INPH}\} \in \{Ra_{d_{i,INPH}}, Rb_{d_{i,INPH}}, Rc_{d_{i,INPH}}, Re_{d_{i,INPH}},$$
$$Rf_{d_{i,INPH}}, Rg_{d_{i,INPH}}, Rh_{d_{i,INPH}}, Rk_{d_{i,INPH}}, Rl_{d_{i,INPH}},$$
$$Rm_{d_{i,INPH}}, Rn_{d_{i,INPH}}, Ro_{d_{i,INPH}}, Rp_{d_{i,INPH}}, Rq_{d_{i,INPH}}, Rr_{d_{i,INPH}}, Rs_{d_{i,INPH}}, Rt_{d_{i,INPH}}, Ru_{d_{i,INPH}}\},$$
$$d_i \in \{d_1, d_2, d_3, \dots\dots\dots d_i \dots\dots., d_N\} \quad \text{(S9)}$$

Section S2

Section S2.1. Mathematical Model of CNN used for multi-class classification and multi-output regression

The convolutional neural network (CNN) is one of the basic building blocks of deep learning [4]. A CNN consists of feature-extraction and classification layers. The feature-extraction layer consists of n number of convolutional and pooling layers. The classification layer is composed of fully connected and output layers. The convolutional layer performs the convolution operation between the input and the kernel to produce the feature map. The output of the convolutional layer is given by:

$$Y_{mn}^{(k)} = f\left(\sum_{u=0}^{L} \sum_{v=0}^{L} H_{uv}^{(k)} X_{mn} + B_{mn}\right) \quad \text{(S10)}$$

where $Y_{mn}^{(k)}$ represents the output feature map, X_{mn} represents the 2D input image, $H_{uv}^{(k)}$ represents the kernel values, k represents the number of kernels, L represents the kernel size, f represents the ReLU activation function, and B_{mn} represents the bias [5]. The output of the convolutional layer was passed through the pooling layer to reduce the dimensionality of the feature map. The output of the pooling layer is given by:

$$Y_{mn} = \max\left(X_{mn}\right) \quad \text{(S11)}$$

where X_{mn} represents the 2D input and Y_{mn} represents the pooling output [5]. The output of final pooling layer was flattened and given to the fully connected layer. The output of the fully connected layer is given by:

$$Y_m = f\left(\sum_{q=1}^{n} W_{vu} X_q + B_m\right) \quad \text{(S12)}$$

where Y_m denotes the output of the fully connected layer, f denotes the ReLU activation function, W_{vu} denotes the weight values, X_q denotes the 1D data obtained through the flattened layer, B_m represents the bias, and n denotes the number of neurons [5]. The output of the fully connected layer was given to the output layer to perform the multi-class classification and multi-output regression tasks. For the multi-class classification, the softmax activation function was used in the output layer; for the multi-output regression, the linear activation function was used. The expression for the softmax function is given by:

$$Y_k = \frac{\exp\left(X_k\right)}{\sum_{q=1}^{n} \exp\left(X_q\right)} \quad \text{(S13)}$$

where Y_k represents the output, X_k represents the input, and n represents the number of neurons [5]. The expression for the linear function is given by:

$$Y = X \quad \text{(S14)}$$

where Y and X represent the output and input values, respectively. [3].

Section S2.2. Performance Metrics

The performance metrics that were considered to evaluate the performance of the multi-class classification task were accuracy, precision, recall, and F1-score [1]. The accuracy can be defined as the proportion of correct predictions to the total number of both correct predictions and wrong predictions [1]:

$$Accuracy = \frac{True\ Positive\ (TP) + True\ Negative\ (TN)}{True\ Positive\ (TP) + True\ Negative\ (TN) + False\ Posive\ (FP) + False\ Negative\ (FN)} \quad \text{(S15)}$$

The precision can be defined as the proportion of the number of correct positive examples to the sum of the correct and wrong positive examples [1]:

$$Precision = \frac{True\ Positive\ (TP)}{True\ Positive\ (TP) + False\ Positive\ (FP)} \quad \text{(S16)}$$

The recall can be defined as the proportion of the number of correct positive examples to the sum of correct positive, and wrong negative examples [1].

$$Recall = \frac{True\ Positive\ (TP)}{True\ Positive\ (TP) + False\ Negative\ (FN)} \quad \text{(S17)}$$

The F1-score can be defined as the proportion of twice the number of correct positive examples to the total sum of twice the number of correct positive examples, wrong positive examples, and wrong negative examples [1]:

$$F1 - score = \frac{2 * True\ Positive\ (TP)}{2 * True\ Positive\ (TP) + False\ Positive\ (FP) + False\ Negative\ (FN)} \quad \text{(S18)}$$

Similarly, the three metrics that were considered to evaluate the performance of the five-class regression task were the mean absolute error (MAE), R^2 score (coefficient of determination), and explained-variance (EV) regression score [3]. The MAE is the absolute difference between the ground truth and the output values:

$$MAE(g, o) = \frac{1}{N} \sum_{i=1}^{N} |g_i - o_i| \quad \text{(S19)}$$

where g_i denotes the ground truth and o_i denotes the output values [3]. The MAE range is not fixed, but the MAE should still be very minimal; i.e., it should approach zero (0). The R^2 score and EV regression score evaluate the performance of a model regarding how well it can predict the future instances. An R^2 score of 1.00 shows that a model has an excellent value, and a score of 0.00 tells us that the model has a consistent value. The expressions for the EV regression score and R^2 score are shown in Eqns. (S20) and (S21), respectively [3]:

$$EV(g, o) = 1 - \frac{Var\{g - o\}}{Var\{g\}} \quad \text{(S20)}$$

where $Var\{.\}$ represents the variance.

$$R^2(g, o) = 1 - \frac{\sum_{i=1}^{N} (g_i - o_i)^2}{\sum_{i=1}^{N} (g_i - g_{bar})^2} \quad \text{(S21)}$$

where $g_{bar} = \frac{1}{N} \sum_{i=1}^{N} (g_i)$ represents the mean or average.

Section S2.3. General five-class confusion matrix

The general multi-class confusion matrix is shown in Figure S3.

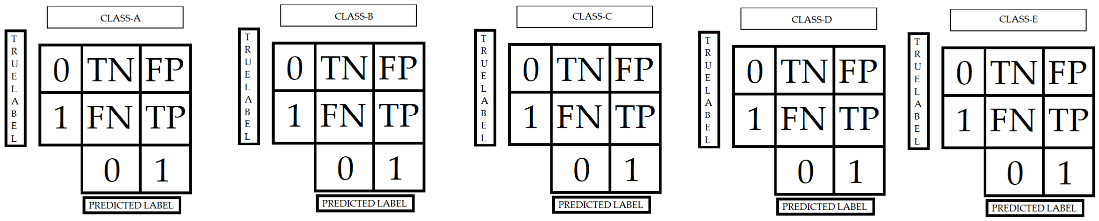

Figure S3. General multi-class confusion matrix.

In Figure S3, it can be observed that the confusion matrix represented five classes; i.e., Class-a, Class-b, Class-c, Class-d, and Class-e. For any class, when the true label and the predicted label were zero, then the condition was called True Negative (TN). When the true label was zero and the predicted label was one, then the condition was called False Positive (FP). When the true label was one and the predicted label was zero, then the condition was called False Negative (FN). Finally, when both the true label and predicted label were one, then the condition was called True Positive (TP). The evaluation metrics for the five-class classification task (accuracy, precision, recall, and F1-score) were calculated for each class; i.e., Class-a, Class-b, Class-c, Class-d, and Class-e using the respective Equations (S15) through (S18). Similarly, the performance metrics for the five-class regression task are obtained by using Equations (S19) through (S21).

1. B. Lokesh Reddy, R N Uma Mahesh & Anith Nelleri (2022), "Deep convolutional neural network for three-dimensional objects classification using off-axis digital Fresnel holography", Journal of Modern Optics, 69:13, 705-717, DOI: 10.1080/09500340.2022.2081371.
2. Michael Liebling, Thierry Blu, and Michael Unser, "Complex-wave retrieval from a single off-axis hologram", J. Opt. Soc. Am. A 21 (3), pp. 367-377, (2004). https://doi.org/10.1364/JOSAA.21.000367.
3. Mahesh, R.N. and Nelleri, A., (2022), "Deep convolutional neural network for binary regression of three-dimensional objects using information retrieved from digital Fresnel holograms", *Applied Physics B*, *128*(8), pp.1-10. https://doi.org/10.1007/s00340-022-07877-w.
4. Y. Lecun, L. Bottou, Y. Bengio and P. Haffner, "Gradient-based learning applied to document recognition," in *Proceedings of the IEEE*, vol. 86, no. 11, pp. 2278-2324, Nov. 1998, doi: 10.1109/5.726791.
5. Tomoyoshi Shimobaba, Naoki Kuwata, Mizuha Homma, Takayuki Takahashi, Yuki Nagahama, Marie Sano, Satoki Hasegawa, Ryuji Hirayama, Takashi Kakue, Atsushi Shiraki, Naoki Takada, and Tomoyoshi Ito, (2017), "Convolutional neural network-based data page classification for holographic memory", Appl.Opt.,56(26),pp.7327-7330.https://doi.org/10.1364 /AO.56.007327.
